# Standardized Workflows for Time-Resolved Singlet Oxygen Quantification in Aqueous Systems

**DOI:** 10.1021/jacsau.5c01463

**Published:** 2026-01-30

**Authors:** Heryerli Fernandez, Helena C. Junqueira, Lucas F. S. Hess, Marcos V. S. Sales, Amanda C. Pinheiro, Carine Arruda, Divinomar Severino, Steffen Hackbarth, Frank H. Quina, Andrés H. Thomas, Carolina Lorente, Maurício S. Baptista, Erick L. Bastos

**Affiliations:** † Department of Biochemistry, Institute of Chemistry, 28133 University of São Paulo, 05508-000 São Paulo, SP, Brazil; § Department of Fundamental Chemistry, Institute of Chemistry, 28133 University of São Paulo, 05508-000 São Paulo, SP, Brazil; ∥ Instituto de Investigaciones Fisicoquímicas Teóricas y Aplicadas (INIFTA), Departamento de Química, Facultad de Ciencias Exactas, Universidad Nacional de La Plata, CCT La Plata-CONICET, Diagonal 113 y 64, S/N, 1900 La Plata, Argentina; ⊥ 9373 Humboldt-Universität zu Berlin, Mathematisch-Naturwissenschaftliche Fakultät, Institut für Physik, Newtonstraße 15, Unter den Linden 6, 10099 Berlin, Germany

**Keywords:** singlet oxygen, phosphorescence lifetime, quantum yield, liposomes

## Abstract

Direct time-resolved phosphorescence detection enables rigorous quantification of singlet oxygen (^1^O_2_, ^1^Δ_g_), yet determining reliable photophysical parameters in aqueous environments remains challenging due to rapid solvent quenching and the kinetic coupling between ^1^O_2_ and photosensitizer (PS) triplet decays, characterized by similar lifetimes (τ_Δ_ ≈ τ_T_). Here we establish standardized workflows for the acquisition and analysis of ^1^O_2_ kinetics in homogeneous and heterogeneous aqueous systems, implemented through the open-source SOLIS computational framework. SOLIS applies homogeneous and diffusion-coupled kinetic models to determine quantum yields and lifetimes, perform structured artifact and model-consistency checks, and quantify lipid-to-water signal contributions using objective fit-quality criteria. Benchmarking with reference photosensitizers demonstrates that this workflow mitigates inconsistencies arising from fitting window selection and provides reliable, self-consistent photophysical parameters. By transforming subjective fitting into a defined practical routine protocol, this approach enhances reproducibility and supports quantitative evaluation of oxidative processes across diverse fields, from photodynamic therapy and polymer degradation to chemical synthesis and environmental science.

## Graphical abstract

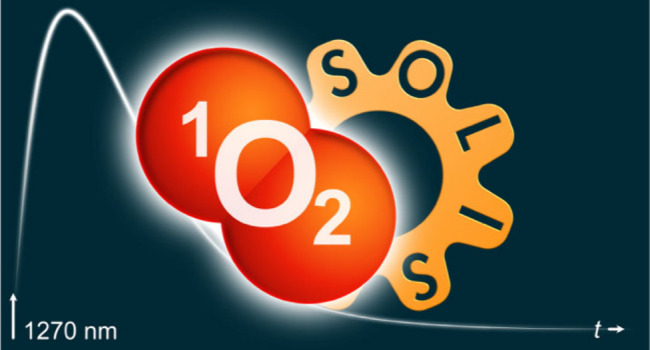

## 1 Introduction

Accurate quantification of the quantum yield (Φ_Δ_) and lifetime (τ_Δ_) of singlet oxygen (^1^O_2_,^1^Δ_g_; see Note S1 for the conceptual basis) is essential for predicting and controlling oxidative processes across photochemical, photobiological, environmental, and technological contexts.^1^ However, even for widely used photosensitizers, reported Φ_Δ_ values in water show method-dependent variation. For example, Rose Bengal is commonly assumed to have Φ_Δ_ ≈ 0.76,^2^ yet values approaching 0.86 have been reported under similar conditions;^3^ TMPyP shows Φ_Δ_ values of ∼0.61 in water^4^ and up to ∼0.74 in deuterated buffer.^2,5^ Discrepancies become more pronounced when comparing across measurement techniques, as illustrated by the case of the flavoprotein miniSOG, for which Φ_Δ_ was reported as 0.47 using chemical trapping^6^ but reassessed as 0.03 by direct time-resolved phosphorescence detection.^7^ These variations underscore the need for a unified workflow that standardizes data acquisition, model selection, and fit-quality assessment to ensure reproducible and mechanistically interpretable ^1^O_2_ parameters.

The sensitivity of ^1^O_2_ quantification to methodological choices arises from the competitive nature of the photosensitization mechanism (Figure 1 and Note S2). After photon absorption and intersystem crossing,^8^ the electronically excited triplet photosensitizer (^3^PS*) transfers energy to ground-state molecular oxygen (^3^O_2_,^3^Σ_g_^–^) to produce ^1^O_2_.^9,10^ Rapid solvent quenching renders the ^1^O_2_ phosphorescence signal inherently weak, with τ_Δ_ in air-saturated water shortened to ∼3.5 μs (20 – 25 °C).^1,11–16^ When τ_Δ_ and τ_T_ become comparable, nonlinear fitting cannot unambiguously separate their contributions, because multiple parameter sets reproduce the same decay profile (Note S3). As a result, the extracted photophysical constants are highly sensitive to fitting-window selection, initial parameter values, and the treatment of short-time artifacts (STAs) arising from detector response or scattered light (Note S4).^2,5,17–19^ Neglecting these factors can produce parameter sets that appear statistically acceptable yet do not reflect the underlying photophysics.

**Figure 1:**
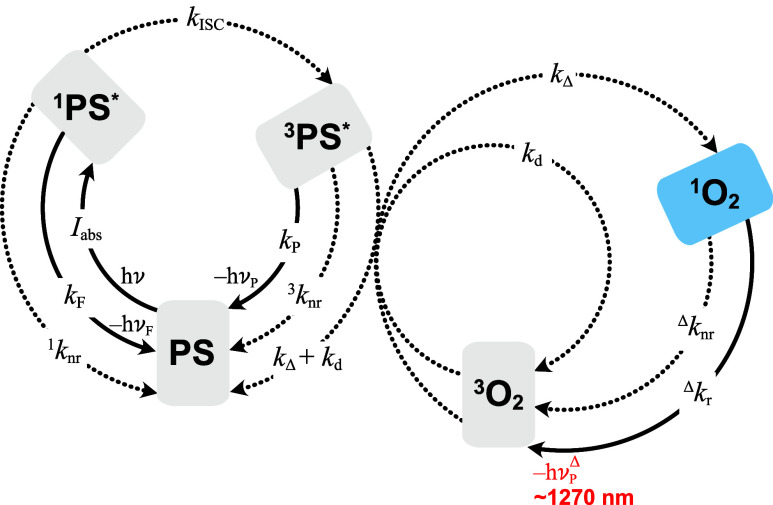
Rate constants relevant to photosensitized ^1^O_2_ generation in the absence of quenchers other than ^3^O_2_. Dotted arrows indicate nonradiative (nr) processes. *I*_abs_ is the rate of photon absorption; rate constant definitions: *k*_F_, ^1^PS* fluorescence, ^1^ *k*_nr_, ^1^PS* nonradiative deactivation, *k*_ISC_, S_1_ → T_1_ intersystem crossing, *k*_P_, ^3^PS* phosphorescence, ^3^ *k*_nr_, ^3^PS* nonradiative deactivation, *k*_Δ_, bimolecular ^1^O_2_ formation via energy transfer, *k*_d_, bimolecular quenching, ^Δ^ *k*_r_, radiative deactivation of ^1^O_2_ via ∼1270 nm phosphorescence, and ^Δ^ *k*_nr_ nonradiative deactivation of ^1^O_2_.

In heterogeneous aqueous environments, including interfaces, vesicles, and cellular systems, additional complexity arises from phase-dependent quenching, nonuniform photosensitizer distribution, spatially varying oxygen availability, and diffusion-coupled generation and decay of ^1^O_2_.^15,16,20–22^ Under such conditions, homogeneous kinetic models systematically obscure early membrane-associated components, distort τ_Δ_ estimates, or attribute diffusion-related features to instrumental artifacts. Diffusion-coupled kinetic modeling (Note S2) resolves these effects by explicitly incorporating interfacial transport and phase-specific decay constants, enabling determination of parameters such as τ_T_, τ_Δ,W_, and the lipid-to-water signal weighting (*A*/*B*).^15,16,20^ Although earlier kinetic models have reproduced rise-decay behavior of ^1^O_2_,^12–16,23–26^ no prior framework has unified heterogeneous-model selection, artifact screening, and parameter validation into a practical workflow accessible to nonspecialists.

To address these challenges, we introduce a standardized, end-to-end workflow for acquisition and analysis of ^1^O_2_ kinetics in both homogeneous and heterogeneous aqueous systems. This workflow is implemented in the open-source Singlet Oxygen Luminescence Investigation Software (SOLIS), which integrates homogeneous and diffusion-coupled kinetic models, performs structured artifact and model-consistency checks, and guides model selection through objective fit-quality criteria. By automating simulation, parameter scanning, and consistency evaluation, SOLIS makes diffusion-based analysis practical for routine use. The workflow also provides conceptual guidance on when homogeneous models are appropriate and when diffusion-coupled models become necessary, a key limitation in prior analytical approaches. The combined protocol-software framework distinguishes valid kinetic parameters from artifacts, ensures cross-study comparability, and provides phase-resolved insight into membrane-associated ^1^O_2_, transforming ^1^O_2_ quantification from a user-dependent fitting exercise into a rigorous and reproducible analytical practice.

## 2 Standardized Protocols

### 2.1 Instrumentation and Analysis Software

Time-resolved ^1^O_2_ measurements were conducted using a time-correlated multiphoton counting system (TCMPC-1270, SHB Analytics GmbH), employing either an embedded pulsed LED array (400, 461, or 632 nm) or an external Nd:YAG laser (532 or 664 nm) as an excitation source (Figure S1), following the methods described in the Supporting Information. All data were analyzed using SOLIS, which implements both homogeneous^2,13,19,24,27–29^ and diffusion-controlled^15,16,20^ kinetic models (see Note S2 for complete mathematical derivation). SOLIS is a multiplatform application, and both the software and instructions for its use are available at https://github.com/el-bastos/SOLIS.

### 2.2 Relative Singlet Oxygen Quantum Yields in Aqueous Solution

The standardized workflow, including operational steps and decision points, to determine Φ_Δ_ of pure photosensitizers in aqueous solution using time-resolved NIR phosphorescence is presented in Figure 2. The following notes supply the conceptual and procedural details omitted from the figure for clarity.

**Figure 2:**
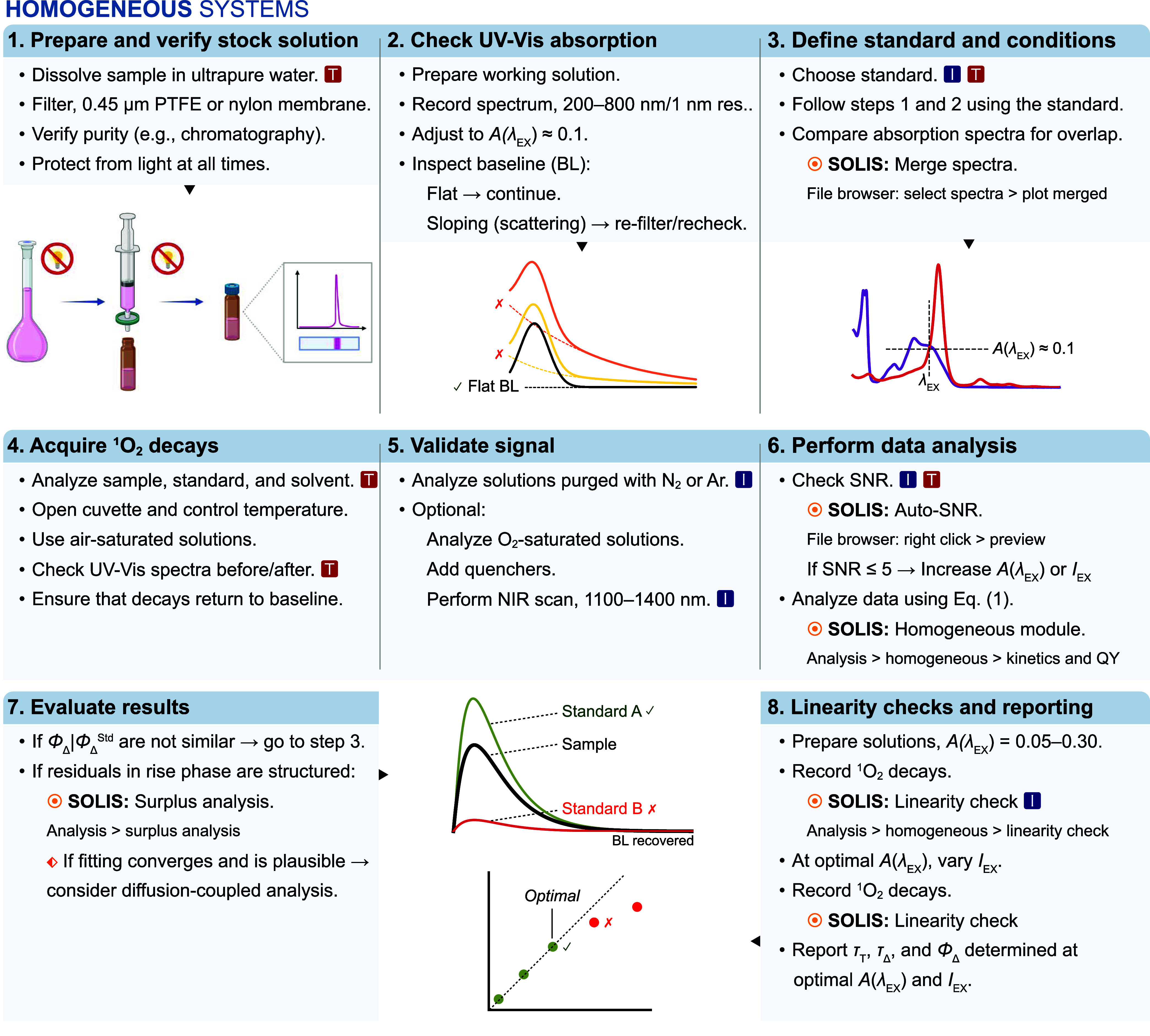
Workflow for determining Φ_Δ_ in aqueous solution using time-resolved NIR phosphorescence spectroscopy. The red box symbol denotes the availability of troubleshooting information in the SI. The blue box symbol highlights critical decision points.

#### 2.2.1 Notes for Homogeneous Systems

**Step 1 –** Prepare stock solutions in ultrapure water (see Note S5 for comments on the use of buffers and DMSO), protect the solutions from light and filter them through 0.45 μm PTFE or Nylon membranes. Record UV–vis absorption spectra over a range that includes at least one nonabsorbing region to assess baseline noise (Notes S4 and S6). Select a standard whose absorption band overlaps the sample and has a similar shape at the chosen excitation wavelength λ_EX_ (Table S1).

**Step 2 –** Adjust both sample and standard to *A*(λ_EX_) ≈ 0.1 (or the lowest value giving peak SNR ≥ 5) and collect ^1^O_2_ phosphorescence decays in air-saturated solution with the cuvette open and temperature controlled. Fit preliminary ^1^O_2_ decays with eq 1, which is implemented in the homogeneous module of SOLIS, and select as standard a PS whose Φ_Δ_ most closely matches that of the sample under identical conditions, so that the corresponding ^1^O_2_ signal amplitudes α are of similar magnitude. If the fits show structured residuals or poor convergence, typically due to early time artifacts, perform control experiments and surplus analysis (Note S7).

$$S\left(t\right)=\alpha \frac{\tau_{\Delta }}{\tau_{\Delta }-\tau_{T}}\left(e^{-\left(t-t_{0}\right)/\tau_{\Delta }}-e^{-\left(t-t_{0}\right)\tau_{T}}\right)+y_{0},t_{0}\geq 0$$ 1

**Step 3 –** Using a series of solutions with *A*(λ_EX_) **≈** 0.05–0.30, verify that α is proportional to the absorbed fraction of incident light, 1–10^–A(λ_EX_)^. At a fixed *A*(λ_EX_), verify proportionality of α with excitation intensity and identify upper limits that avoid saturation and photobleaching. These tests define the optimal *A*(λ_EX_) and excitation settings for quantitative Φ_Δ_ determination.

**Step 4 –** Under validated conditions and matched *A*(λ_EX_), determine α for sample and standard (Std) and calculate Φ_Δ_ via eq 2:

$$\Phi_{\Delta }=\Phi_{\Delta }^{\mathrm{Std}}\frac{\alpha }{\alpha^{\mathrm{Std}}}\frac{1-10^{-A{\left(\lambda_{\mathrm{EX}}\right)}^{\mathrm{Std}}}}{1-10^{-A\left(\lambda_{\mathrm{EX}}\right)}}$$ 2

where Φ_Δ_^Std^ is taken from the literature (Table S1). Matching *A*(λ_EX_) minimizes propagated uncertainties.

### 2.3 Singlet Oxygen Lifetimes in Liposomes

The workflow depicted in Figure 3 outlines the operational sequence used to determine τ_T_, τ_Δ,W_, and the aqueous-lipid rate ratio (*A*/*B*) for PSs associated with liposomes.

**Figure 3:**
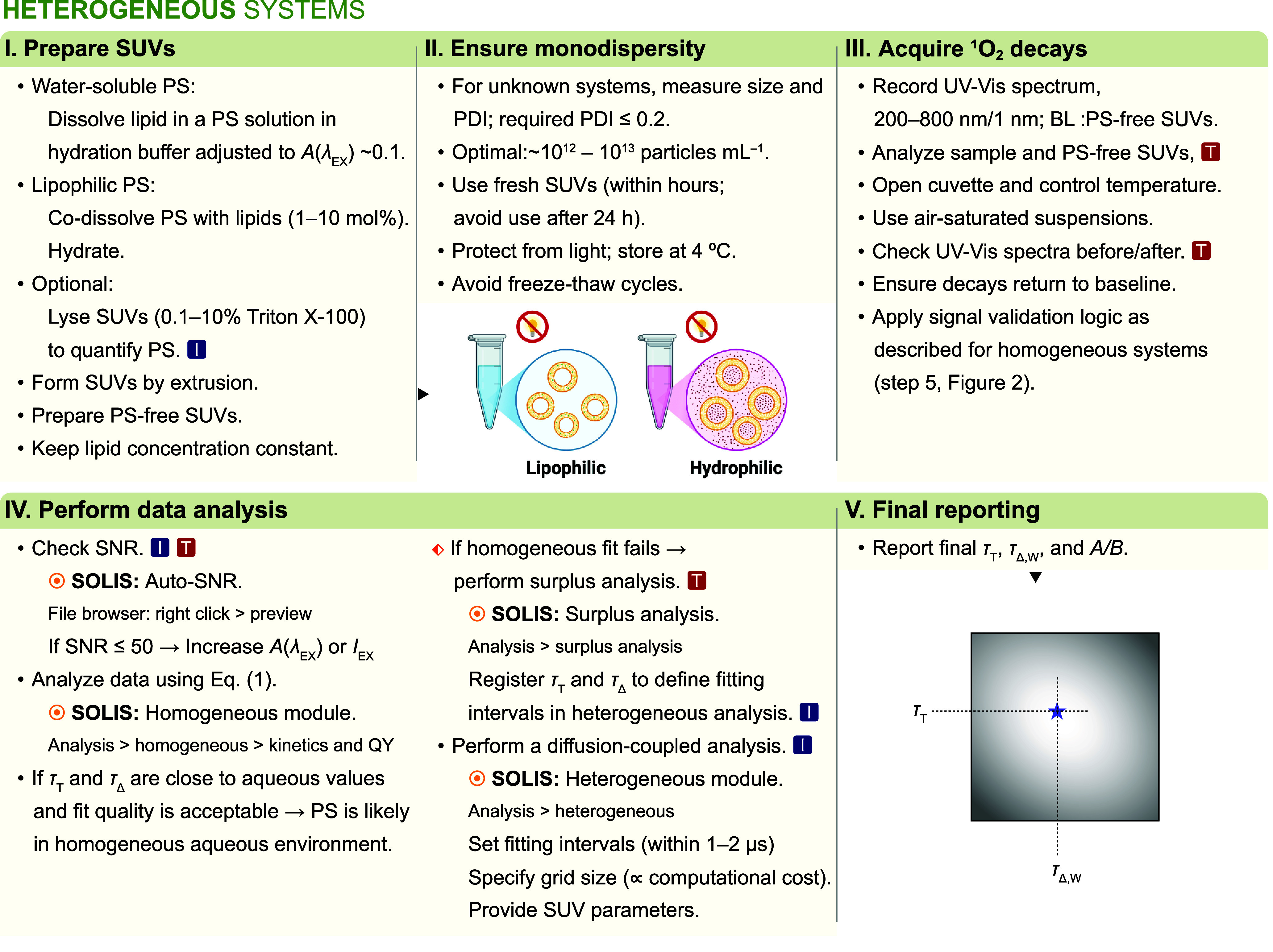
Workflow for the determination of singlet oxygen quantum yields in aqueous liposome suspensions using time-resolved NIR phosphorescence spectroscopy. The red box symbol denotes the availability of troubleshooting information in the SI. The blue box symbol highlights critical decision points.

**Steps I and II –** Prepare small unilamellar vesicles (SUVs) of the desired composition and verify size and polydispersity (PDI < 0.2) by DLS when required. Prepare SUVs with and without photosensitizer; depending on the goal, use standards with different log P values (e.g., Pheophorbide a, TMPyP). Perform all measurements using fresh vesicles.

**Step III –** Record UV–vis absorption spectra using PS-free SUVs for baseline correction and/or perform vesicle lysis to estimate PS content. Choose *A*(λ_EX_) at or near the maximum PS absorption in the suspension. Acquire ^1^O_2_ decays at 1270 nm in air-saturated suspensions (cuvette open), targeting SNR ≥ 50. When available, collect auxiliary decays at 1250 and 1300 nm for artifact suppression.

**Step IV –** Fit ^1^O_2_ signal with eq 1 in SOLIS. Agreement of τ_Δ_ with reference aqueous values (≈3.5 μs in water, ≈4.1 μs in 7% EtOH­(aq)) indicates a predominantly aqueous/interfacial environment. If the homogeneous model fails to describe the kinetics (e.g., structured residuals, inconsistent τ_Δ_), perform surplus analysis (Note S7) and, if early decay complex kinetic behavior is observed, fit the data using the diffusion-reaction model [eq 3] implemented in the Heterogeneous module of SOLIS. This yields τ_T_, τ_Δ,W_, and the aqueous-lipid rate ratio *A*/*B*; fixed parameters (e.g., membrane thickness, τ_Δ,L_) and fitting ranges (see Note S2).

$$S\left(t\right)=A\cdot n_{L}\left(t\right)+B\cdot n_{W}\left(t\right)+C$$ 3

## 3 Results and Discussion

### 3.1 Aqueous Solutions: Homogeneous Systems

This section outlines a comprehensive workflow for the presentation and fitting of kinetic data, illustrating through practical examples how experimental design, model selection, and data analysis window critically influence the extracted parameters (τ_T_, τ_Δ_, and Φ_Δ_). To ensure methodological robustness and reproducibility, we use well-known water-soluble photosensitizers, such as TMPyP,^30^ 1*H*-phenalen-1-one (phenalenone, PN),^31,32^ Rose Bengal (RB),^3,33^ under experimental conditions chosen to highlight scenarios that commonly lead to errors or misinterpretation of results.

#### 3.1.1 Presenting and Fitting Kinetic Data

Kinetic traces of ^1^O_2_ phosphorescence exhibit an exponential rise followed by a decay and are displayed after removal of the initial baseline signal (IBL) and short-time artifacts (STAs) (Figures 4a–**b**). In air-equilibrated H_2_O at room temperature, the signal returns to baseline within ∼50 μs owing to the short lifetime of ^1^O_2_ (τ_Δ_ = 3.5 μs), whereas in D_2_O the observation window must be extended (τ_Δ_ = 66 ± 2 μs, Figure S2).^34^

**Figure 4:**
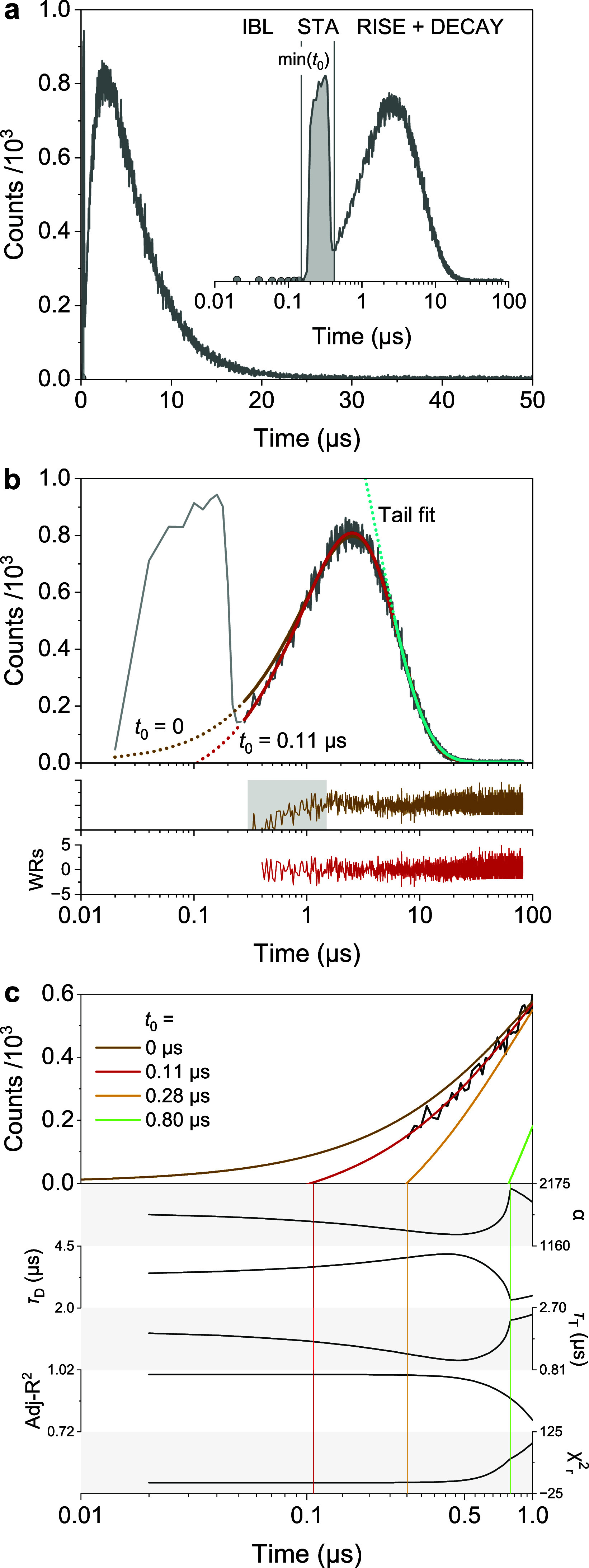
Kinetic traces of ^1^O_2_ (^1^Δ_*g*_) phosphorescence and corresponding model fits in air-saturated water, illustrating data presentation, scaling, and fitting approaches. (a) Raw experimental data for PN excited at 400 nm with a pulsed LED; the inset shows the same data on a log_10_ time scale to better visualize and distinguish each phase of the temporal profile. (b) Processed kinetic trace of PN with the IBL and STA region removed and the time origin (*t* = 0) defined as the onset of the excitation pulse. Fits using eq 1 are shown with *t*_0_ fixed at zero (orange) or included as a fitting parameter (red), along with corresponding weighted residuals. The gray area shows the region of concern. (c) Effect of *t*_0_ on values of α, τ_Δ_, τ_T_, Adj-R^2^, and χ^2^_r_.

To determine τ_T_, τ_Δ_, and the amplitude α, eq 1 is fitted to the experimental ^1^O_2_ signal, *S*(*t*),^35^ provided that both rise and decay contain sufficient points. This condition is met when the peak signal-to-noise ratio (SNR) is at least 5:1 and the STA duration is much shorter than the rise time (Figure S2). The model also fits *t*_0_ (≥0) and *y*_0_, which account for the onset of detectable ^1^O_2_ formation and any baseline offset, respectively. In routine analyses, *t*_0_ should be treated as a free parameter unless the STA is negligible relative to the rise time and the onset of ^1^O_2_ formation is unambiguously resolved. Fixing *t*_0_ = 0 is only appropriate when the STA has fully decayed before the rise begins; otherwise, forced alignment artificially distorts τ_T_ or τ_Δ_ and reduces reproducibility (Figures, S3, and S4). Evaluation of residuals and reduced χ^2^ determines whether freeing *t*_0_ is warranted. Unlike fluorescence decays, where only the exponential tail is used, the rise phase, when present, provides essential information for separating τ_T_ and τ_Δ_. For this reason, plotting time on a logarithmic scale improves visualization and fitting robustness (Figure 4a).

For TMPyP, fits with fixed or free *t*_0_ yield identical parameters (Figure S3). For PN, however, freeing *t*_0_ decreases τ_T_ by ∼15% and increases τ_Δ_ by ∼9%, illustrating that defining the onset at the STA boundary leads to systematic deviations in early time residuals and altered χ^2^_r_ values (Figures 4b–c). Using PN (Φ_Δ_ = 0.98 ± 0.08)^32^ as standard, the Φ_Δ_ of TMPyP at 400 nm is 0.76 ± 0.07, consistent with literature (0.74).^2^ This contrast between PN and TMPyP underscores that fitting choices can bias τ_T_ and τ_Δ_ even when Φ_Δ_ remains consistent, which is critical for studies emphasizing kinetics.

The wavelength independence of Φ_Δ_ was verified for TMPyP at 450 nm (Φ_Δ_ = 0.73 ± 0.07), with similar results for riboflavin (0.56 ± 0.06 vs literature value:^36^ 0.58 ± 0.06) (Figure S3d). Tail-only fits produced longer apparent τ_Δ_ values (4.2 ± 0.4 μs) than full trace fits (3.42 ± 0.05 μs), matching the expected value (3.5 μs).^13–15^ Tail fitting is therefore not recommended,^37^ as restricting analysis to the decay phase couples parameters and reduces reproducibility (Figures 4b, S3d, and S5). Accurate τ_Δ_ determination requires fitting the full processed trace with a physical rise-decay model. Independent τ_T_ measurements by transient absorption can further constrain the fit.^21,38^

Manual analysis of ^1^O_2_ traces is labor-intensive and prone to user-dependent selection of fitting windows (Figure S4). To minimize bias, all data sets were analyzed using SOLIS, which automates STA identification, SNR evaluation, fitting-window selection, nonlinear regression, and Φ_Δ_ calculation with full error propagation. Example data sets are provided within SOLIS for reproducibility.

#### 3.1.2 Linearity Check and Spectral Validation of the ^1^O_2_ Signal

The SNR is one of the most critical parameters governing the accuracy of ^1^O_2_ phosphorescence measurements. Achieving SNR ≥ 5 depends on detection sensitivity, Φ_Δ_, and the presence of quenchers.^37,39^ Increasing PS concentration or excitation intensity (*I*_EX_) can improve SNR but may induce sample heating, aggregation, detector saturation, or unwanted photophysical and photochemical transformations such as photobleaching and multiphoton excitation. Inner-filter effects^40^ further distort results by reducing excitation efficiency (primary) and reabsorbing emission (secondary). Together, these effects introduce nonlinearities that compromise the reliability of α, Φ_Δ_, and τ_Δ_.

For quantitative work, the absorbance at the excitation wavelength, *A*(λ_EX_), should be low yet sufficient to generate measurable ^1^O_2_ emission. Under linear conditions, α is proportional to the absorbed fraction of incident light, 1–10^–A(λ_EX_)^. Significant deviations or nonzero intercepts in α versus 1–10^–A(λ_EX_)^ plots typically indicate instrumental offsets or background luminescence, whereas curvature or saturation suggests scattering, impurities, or early aggregation (Note S8). To illustrate, TMPyP solutions irradiated at 400 nm show linear α up to *A*(λ_EX_) = 0.3 (4.2 μmol L^–1^); above this, deviations mark the onset of inner-filter and aggregation effects (Figure 5a). The extent of linearity is PS-dependent and must be established experimentally. Consistent mean τ_Δ_ values across concentrations (here 3.5 ± 0.2 μs) confirm that the linear range reflects intrinsic photophysics rather than concentration artifacts.

**Figure 5:**
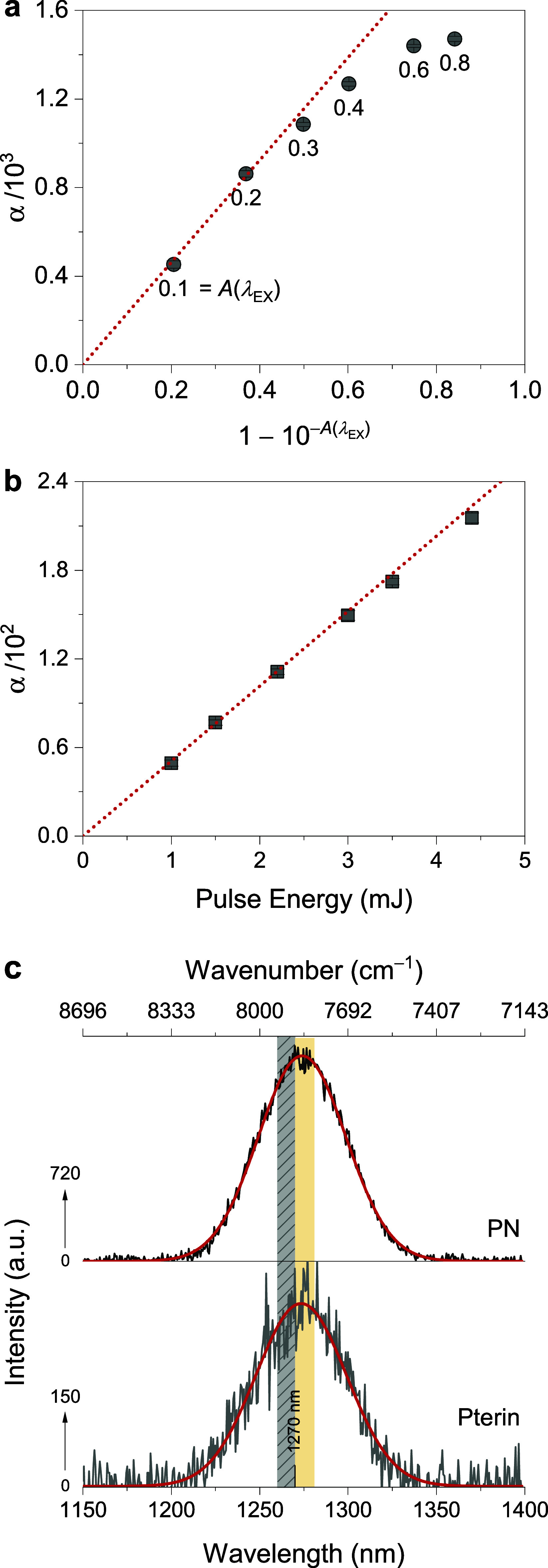
Effect of absorbance and excitation power on the amplitude parameter α and singlet-oxygen emission spectra. (a) Dependence of α on the absorbance of TMPyP in air-saturated H_2_O; 400 nm. Solid lines are the fits to eq 1; gray shading indicates 1σ (*N* = 7). Dependence of α on excitation power for (b) Rose Bengal; red lines are through-origin regressions, gray shading indicates 1σ (*N* = 3). Residuals in the STA region were intentionally left uncropped to highlight discrepancies at this limit. Raw data is presented in Figure S6. (c) NIR emission spectra of PN and pterin in D_2_O; gray region (1260–1270 nm) marks the “zone of deception” and green region (1270–1281 nm) the “zone of confidence”; red curves are Gaussian fits.

Optimizing excitation conditions is equally important. Even with a sensitive detector, the excitation must remain low enough to avoid photobleaching, triplet–triplet annihilation, or nonlinear optical processes, while maintaining adequate SNR.^23,37–39^ Such effects are diagnosed by deviations from linearity between excitation intensity and α. For Rose Bengal, α scales linearly with pulse energy up to ∼4 mJ (Figure 5b); deviations at higher energy identify the onset of photophysical distortions. When the regression is constrained to pass through the origin, it is unnecessary to compute Φ_Δ_ from the slope of the α versus intensity plot because the signal ratio at any point along the line is identical to the ratio of the slopes.

Even when kinetic traces are well behaved, it is essential to confirm that emission detected at 1270 nm originates from ^1^O_2_ and not from PS phosphorescence or instrumental background. Many PSs emit in the far-red/NIR (650–1100 nm), and long-wavelength tails can overlap the ^1^O_2_ band. These contributions may distort the rise region or mimic multicomponent kinetics. It is therefore advisable to record the NIR phosphorescence spectrum of the PS in deoxygenated solution^41^ as well as in air-saturated conditions before kinetic analysis.

Harvey and co-workers identified a “zone of deception” in the 1260–1270 nm range, where broad background processes, including PS phosphorescence tails, detector afterglow, and scattered excitation light, contribute luminescence that lacks the sharp, environment-invariant signature of ^1^O_2_.^42^ In contrast, the 1270–1281 nm “zone of confidence” corresponds to the well-defined ^1^O_2_(^1^Δg) → O_2_(^3^Σg^–^) transition and provides a reliable spectral window for authentic ^1^O_2_ detection.^42^ Several additional NIR processes, including triplet–triplet annihilation of the PS, ^1^O_2_-sensitized delayed fluorescence, and dimol emission of ^1^O_2_, can contribute weak, broad backgrounds in the 1200–1300 nm region.^43^ Although typically weak and spectrally broad, these signals can distort the early time region sufficiently to bias model selection in borderline cases. Power-dependence tests, O_2_/Ar modulation, and comparison of 1250/1270/1300 nm channels readily distinguish these contributions from authentic ^1^O_2_ emission.

To illustrate, NIR spectra of PN and pterin in air-saturated D_2_O both show maxima at 1273 ± 1 nm, confirming assignment to ^1^O_2_ (Figure 5c).^44,45^ When spectral signatures remain ambiguous, comparing spectra under air, O_2_, and Ar/N_2_ environments is decisive. ^1^O_2_ emission vanishes upon deoxygenation and remains the same or intensifies under O_2_ saturation, whereas background signals are largely insensitive to or partially quenched by oxygen. Neglecting these controls risks misassigning PS emission or detector artifacts as singlet oxygen, leading to fundamentally incorrect Φ_Δ_ or mechanistic conclusions.

#### 3.1.3 Selection of the Standard

Once the authenticity of the ^1^O_2_ signal has been verified, accurate Φ_Δ_ determination requires appropriate selection of a PS standard. A summary of available standards for aqueous media is provided in Table S1. Suitable standards should be available in pure form, thermally and photochemically stable, and fully soluble in water without the need of organic cosolvents such as DMSO (see SI for details). Their absorption spectra must overlap with the excitation wavelengths and resemble that of the analyte to minimize wavelength-dependent uncertainties. Representative absorption spectra and Φ_Δ_ values for common standards in water are shown in Figure S7. The raw spectral data sets are deposited in the PhotochemCAD database.^46,47^

An appropriate standard must ideally also remain nonaggregated at the concentration used to achieve sufficient SNR (see SI for details). Overlooking aggregation can lead to the false conclusion that a PS has an intrinsically low Φ_Δ_ when the apparent decrease arises from concentration-dependent aggregation or improper sample preparation. The Φ_Δ_ of the standard should be reasonably close to that of the sample; large mismatches require disproportionately different integration times or excitation conditions, reducing comparability. For intrinsically low-Φ_Δ_ systems such as dissolved organic matter (DOM), high-efficiency reference sensitizers like PN remain the widely recommended choice. In these cases, the mismatch between sample and standard signal intensities may require adjusting excitation power, averaging routines, and SNR thresholds. Although these practical considerations are not limitations of the reference sensitizer, they can determine the appropriate workflow for low-Φ_Δ_ samples.^23,38^ Finally, if the photophysical properties of the PS are pH sensitive, the pH adjustment must be strictly performed to ensure that a single acid–base species is present during the measurements. The use of organic cosolvents or refractive-index corrections to Φ_Δ_ are not recommended, and *A*(λ_EX_) should be matched as closely as possible between sample and standard.

### 3.2 Liposome Suspensions: Heterogeneous Systems

The kinetic analysis of singlet oxygen in heterogeneous environments, such as liposome suspensions, introduces complexity beyond that encountered in homogeneous aqueous solutions. In these systems, the PS can partition between the aqueous and lipid phases, producing distinct microenvironments for ^1^O_2_ generation and decay. This partitioning also enables analysis of ^1^O_2_ diffusion across the lipid–water interface through τ_Δ_. Accordingly, this section focuses on acquiring reliable phosphorescence kinetics and extracting physically meaningful parameters using homogeneous or diffusion-coupled reaction–diffusion models, with model selection determined directly from the data. A detailed treatment of ^1^O_2_ quenchers as mechanistic controls, including their limitations in heterogeneous systems, is available elsewhere.^15,39^

#### 3.2.1 Photosensitizer Localization and Its Impact on ^1^O_2_ Kinetics

If the PS resides almost exclusively in one compartment of the liposomal suspension, the decay behaves as in a homogeneous medium and is well described by eq 1, which assumes a single diffusion coefficient and lifetime. Hydrophilic sensitizers with small log P values generate ^1^O_2_ predominantly in water [*B* ≫ *A* in eq 3] and the decay remains governed by τ_Δ,W_ ≈ 3.5 μs (in water). Under these conditions, the phosphorescence traces resemble those obtained in homogeneous aqueous solution, and fits yield physically consistent τ_T_ and τ_Δ_ values. This scenario likely explains previous reports of successful homogeneous-model fits in liposomes for water-soluble sensitizers such as TMPyP.^20^ In practice, agreement of τ_Δ_ with its aqueous value, together with structureless residuals, indicates that the homogeneous model remains valid even in the presence of liposomes.

When the PS partitions into the lipid bilayer, the observed ^1^O_2_ kinetics deviate from simple rise–decay behavior, particularly in the early time region, and eq 1 fails to reproduce the rise and initial decay. This deviation reflects the contributions of lipid-phase ^1^O_2_, which exhibits a longer intrinsic lifetime (τ_Δ,L_ ≈ 12–16 μs),^15,16,20^ combined with aqueous-phase ^1^O_2_ and interfacial transport. To account for this heterogeneity, the diffusion-reaction model of Hackbarth and Röder describes the kinetics as the sum of lipid and aqueous contributions, weighted by coefficients *A* and *B* [eq 3 **]**, respectively (see Item 2.2 in SI for the full derivation).^20^ A water-soluble PS such as TMPyP yields fits dominated by the aqueous phase, whereas a more lipophilic PS such as pheophorbide a (Pheo) produces kinetic profiles that clearly require lipid–water coupling. In such cases, the early rise is shaped by lipid-associated ^1^O_2_, and accurate extraction of τ_T_ and τ_Δ,W_ requires the heterogeneous model. When these deviations become evident, the kinetics clearly require transition from the homogeneous formulation to a diffusion-coupled description, as detailed below.

#### 3.2.2 Extracting Physically Meaningful Parameters in Heterogeneous Liposomal Systems

Experimental traces from heterogeneous liposomal suspensions are processed as described for homogeneous systems, but the presence of multiple microenvironments necessitates additional steps. When eq 1 is applied to sensitizers that partition into the membrane, such as Pheo in DPPC (dipalmitoylphosphatidylcholine) vesicles prepared in aqueous ethanol, the fit fails to describe the early rise and the resulting τ_T_ and τ_Δ_ deviate from expected values (≈2 μs and ≈4.1 μs).^20^ These discrepancies, combined with structured residuals, provide a direct indication that lipid-associated ^1^O_2_ contributes measurably to the kinetics and that a diffusion-coupled analysis is required.

To obtain stable initial estimates for the simulation-based fit, the late-time region of the decay (typically *t* > 7 μs) is first fitted using eq 1, which approximates the aqueous-phase ^1^O_2_ component (Figure 6a). The difference between this preliminary fit and the raw trace isolates the additional (surplus) lipid-phase signal, from which approximate rise-related parameters can be extracted using simplified regressions. These values do not constitute final photophysical parameters, but restrict the multidimensional τ_T_|τ_Δ,W_ search space and thereby facilitate convergence of the diffusion-reaction model.

**Figure 6:**
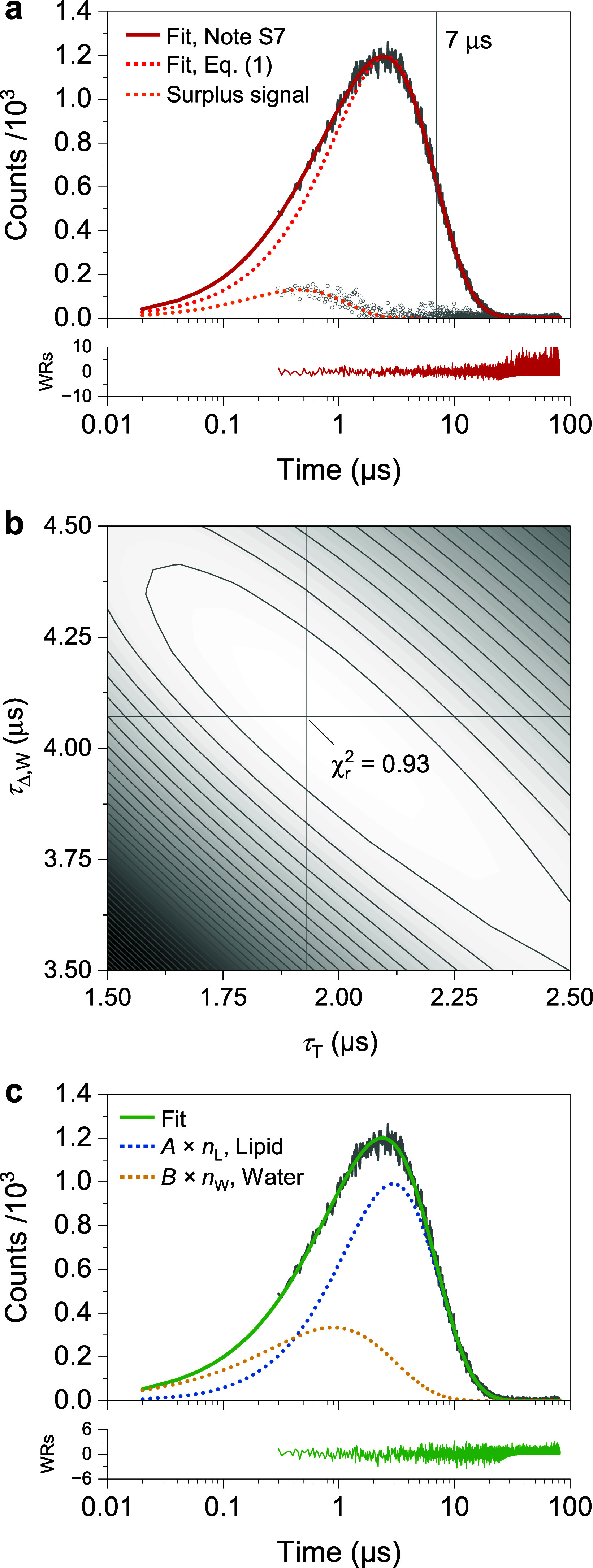
Analysis of a heterogeneous suspension of Pheo in DPPC liposomes. (a) Surplus signal and global fit using eq 3. (b) Contour plot of χ^2^_red_ as a function of τ_T_ and τ_Δ,W_. Each parameter pair was fitted to the data using simulated ^1^O_2_ dynamics to determine the coefficients *A*, *B*, and *C*. (c) Final fit with eq 3 and deconvoluted kinetic traces of ^1^O_2_ in the water and lipid phases.

The full heterogeneous analysis employs the diffusion-reaction model, in which the vesicle is represented as concentric shells comprising lipid and aqueous regions. At each trial pair of τ_T_ and τ_Δ,W_, the coupled diffusion–reaction equations governing transport and decay of ^1^O_2_ in both phases are numerically integrated to generate the basis functions *n*_L_(t) and *n*_W_(t) (see Note S2 for complete derivation).^12,16,17,20^ The experimental signal is modeled as a linear combination of these components, and coefficients *A*, *B*, and *C* of eq 3 are obtained by least-squares regression. The lipid-phase lifetime τ_Δ,L_ is fixed within its known range (12–16 μs), as variations negligibly affect the kinetics relative to interfacial transport.

Application of this method to Pheo in DPPC vesicles yields a χ^2^_red_ surface (Figure 6b) with a well-defined minimum at τ_T_ = 1.93 μs and τ_Δ,W_ = 4.07 μs, in excellent agreement with values independently obtained from transient absorption and aqueous-lifetime measurements (≈2 μs and ≈4.1 μs).^20^ The recovered **A/B** ratio (3.43) confirms that ^1^O_2_ is generated predominantly in the lipid environment. Importantly, **A/B** reflects the relative contribution to the detected signal, not absolute ^1^O_2_ yields per phase. Deconvolution of the simulated components (Figure 6c) shows that the early rise originates from lipid-phase ^1^O_2_, whereas the later decay is dominated by the aqueous component, reflecting diffusion from the bilayer into the surrounding medium. Because the diffusion-controlled model relies on numerical simulation rather than a closed-form expression, it is computationally intensive. SOLIS automates preprocessing, initial parameter estimation, multidimensional scanning of τ_T_ and τ_Δ,W_, and extraction of A/B, enabling routine and reproducible analysis of membrane-associated ^1^O_2_ kinetics without user-dependent fitting choices.

## 4 Conclusions and Outlook

The workflows established here provide a unified, experimentally validated framework for reliable quantification of singlet-oxygen kinetics in aqueous media. By integrating homogeneous and diffusion-coupled models within the open-source SOLIS platform, the approach transforms ^1^O_2_ phosphorescence analysis from a subjective, user-dependent procedure into a standardized and reproducible methodology. The resulting kinetic parameters, τ_T_, τ_Δ_, Φ_Δ_, and lipid–water phase contributions, are obtained with internal consistency checks, clearly defined model-selection criteria, and a data structure that supports cross-laboratory comparability. These workflows directly establish practical, mechanistically grounded protocols for quantitative photochemical studies in aqueous and membrane-mimetic systems.

Looking forward, extending time-resolved ^1^O_2_ detection into living cells and tissues remains an unresolved frontier.^1,21,48–52^ The intracellular environment contains several potential quenchers.^1,50,53^ In addition, it is optically heterogeneous, chemically discontinuous, and dynamically perturbed by illumination, rendering absolute lifetimes and quantum yields neither transferable nor well-defined. As such, cellular ^1^O_2_ measurements should be interpreted as comparative metrics anchored to rigorously characterized extracellular benchmarks such as those established here. Progress toward true intracellular quantification will require simultaneous advances in instrumentation, physically constrained modeling, and biologically defined reference systems, including targeted or genetically encoded photosensitizers with known triplet yields. Ultimately, the conceptual challenge is not merely analytical but foundational: to redefine how photochemical observables relate to reactivity in a nonequilibrium, compartmentalized medium. The workflows presented in this work provide the reference axis against which this complexity can be measured, offering a practical foundation for future efforts to transform ^1^O_2_ luminescence from a qualitative indicator into a quantitative descriptor of oxidative flux in living matter.

## Supplementary Material



## Notes

Materials and methods; Supplementary Figures; Photophysical properties of common water-soluble photosensitizers; Notes; Troubleshooting. (PDF)
