# Supplementary material for: Standardized Workflows for Time-Resolved Singlet Oxygen Quantification in Aqueous Systems
Source: JACS Au. 2026 Jan 30;6(4):2627–36. doi: 10.1021/jacsau.5c01463 (PMC13126201; doi:10.1021/jacsau.5c01463)
Supplement: Supplementary file 1 [file au5c01463_si_001.pdf]

SUPPORTING INFORMATION

Standardized Workflows for Time-Resolved Singlet Oxygen Quantification in Aqueous Systems

Heryerli Fernandez,[a,b,‡] Helena C. Junqueira,[a,c,‡] Lucas F. S. Hess,[a] Marcos V. S. Sales,[a] Amanda C. Pinheiro,[a] Carine Arruda,[a] Divinomar Severino,[c] Steffen Hackbarth,[d] Frank H. Quina,[a] Andrés Thomas,[b] Carolina Lorente,[b] Maurício S. Baptista,[c,*] and Erick L. Bastos[a,*]

[a] Department of Fundamental Chemistry, Institute of Chemistry, University of São Paulo, 05508-000 São Paulo, SP, Brazil.

[b] Instituto de Investigaciones Fisicoquímicas Teóricas y Aplicadas (INIFTA), Departamento de Química, Facultad de Ciencias Exactas, Universidad Nacional de La Plata, CCT La Plata-CONICET, Diagonal 113 y 64, S/N, 1900 La Plata, Argentina

[c] Department of Biochemistry, Institute of Chemistry, University of São Paulo, 05508-000 São Paulo, SP, Brazil.

[d] Humboldt-Universität zu Berlin, Mathematisch-Naturwissenschaftliche Fakultät, Institut für Physik. Newtonstraße 15, Unter den Linden 6, 10099 Berlin

‡ Both authors contributed equally to this work.

* Corresponding authors: baptista@iq.usp.br (MSB) and elbastos@usp.br (ELB).

SUMMARY

Materials and Methods S4

General S4

Absorption Spectra S4

Steady-State Singlet Oxygen Emission S4

Singlet Oxygen Lifetime Instrumentation S5

Mathematical and Statistical Analysis S6

Figures S7

Photophysical Properties of Water-Soluble Photosensitizers S14

Notes S17

Note S1 – A Primer on Singlet Oxygen S17

Note S2 – Models for Kinetic Analysis S18

Homogeneous Systems S18

The Diffusion–Reaction Model S23

Note S3 – Challenges in Non-Linear Curve Fitting S25

Note S4 – Processing Raw Data S28

Baseline Correction S28

Short-Time Artifacts S29

Note S5 – Aqueous Media S30

Buffer Selection S30

Cell Culture Media and DMSO S31

Note S6 – Rigorous Absorbance/Optical Density Measurement........ S33

Note S7 – Surplus Analysis S34

Note S8 – Aggregation of Photosensitizers S35

Troubleshooting S37

References........ S42

MATERIALS AND METHODS

General

All photosensitizers (PSs) used in this work were obtained from commercial sources, except as otherwise noted. Solutions were prepared using deionized water (18.2 MΩ cm at 25 ± 2 ºC, TOC ≤ 4 ppb, Milli-Q, Millipore). All cuvettes were washed thoroughly using an alkaline cleaning concentrate, following the instructions of the manufacturer (Hellmanex III, Hëllma Analytics).

Absorption Spectra

Absorption spectra were acquired in a 10 mm pathlength quartz cuvette using either a Varian Cary 50 Bio or a Shimadzu UV-2400-PC spectrophotometer, both equipped with thermostated cell holders maintained at 25 ± 1 °C. Spectra were collected over the 200 – 800 nm range with a signal averaging time of 12.5 ms, a data interval of 1 nm, and a scan rate of 4,800 nm min^{-1}.

Steady-State Singlet Oxygen Emission

Singlet oxygen emission spectra were acquired at 25 ± 2 ºC using a FLS980 time-resolved photoluminescence spectrometer (Edinburgh Instruments Inc.) equipped with a $N_{2(\ell)}$-cooled R5509-72 photomultiplier (Hamamatsu) and a Q-Smart 850 laser coupled to a Rainbow VIR optical parametric oscillator (OPO) pumped at 355 nm (Quantel, output range: 410–680 nm, repetition rate: 10 Hz, pulse duration ~6 ns, pulse energies up to 5 mJ). Spectra of air-equilibrated aqueous PS solutions were collected over the 1,150 – 1,400 nm range.

Singlet Oxygen Lifetime Instrumentation

Measurements of $^1O_2(^1\Delta_g)$ phosphorescence were conducted using a TCMPC-1270 Luminescence Detection System (SHB Analytics GmbH).[1, 2] Excitation was provided by either an embedded pulsed LED array (400 nm, 461 nm, or 632 nm; **Figure S1**) or an external Nd:YAG laser (532 nm or 664 nm). Laser experiments were conducted with a measurement time of 100 s and the average power was measured at the entrance of the excitation laser beam into the sample chamber with a LabMax/J 10MT-10 kHz energy sensor (Coherent Inc.). Measurements using the LED module (measurement time: 5 s) were conducted in flat-bottomed cylindrical (⌀10 mm) quartz cuvettes, whereas the laser module used 10 mm optical pathlength quartz cuvettes. Air-equilibrated PS solutions and liposome suspensions were maintained under magnetic stirring at 25 ± 2 ºC. The time-correlated multi-photon counting (TCMPC) setup comprises compact detection optics to collect the emission and collimate it toward the detector. The optics also cover spectral discrimination centered at ~1270 nm (40 nm FWHM) by multi-layer thin film coating. The typical peak transmission is 80% and out-of-band blocking (>10^5) from the UV to >1700 nm. Luminescence was detected with a thermoelectric cooled photomultiplier tube (H10330-45, Hamamatsu/SHB Analytics GmbH, modified version for improved étendue[†]), with spectral response from 950 to 1,400 nm and a quantum efficiency between 2 and 4.5%. The anode pulse rise time (900 ps) and transit time spread (400 ps) were negligible within the $^1O_2$ luminescence time domain. Signal registration was performed with a USB controlled TCMPC counting electronics, which also triggers the excitation source at a repetition rate of 12 kHz. The TCMPC module featured a channel width of 20 ps and a total of 4096 channels (81.92 μs).

[†] Étendue is a fundamental concept in optics that describes the spread of light in both space and angle, representing the light-carrying capacity of an optical system.

Mathematical and Statistical Analysis

Nonlinear regressions and statistical analyses were performed using Origin 2025b (OriginLab) and the open-source Singlet Oxygen Luminescence Investigation Software, SOLIS, available under the Creative Commons Attribution 4.0 (CC BY-NC 4.0) license in the Zenodo and GitHub repositories.

FIGURES

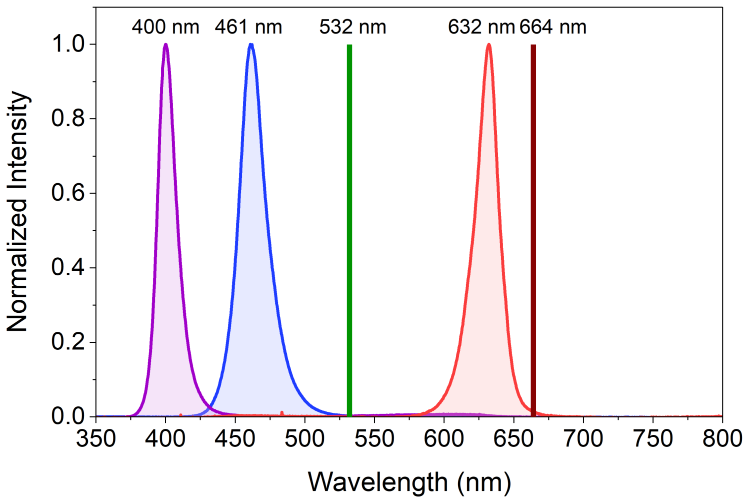

Figure S1. Emission spectra of the pulsed light-emitting diodes (LEDs) employed as excitation sources (maxima at 400, 461, or 632 nm).

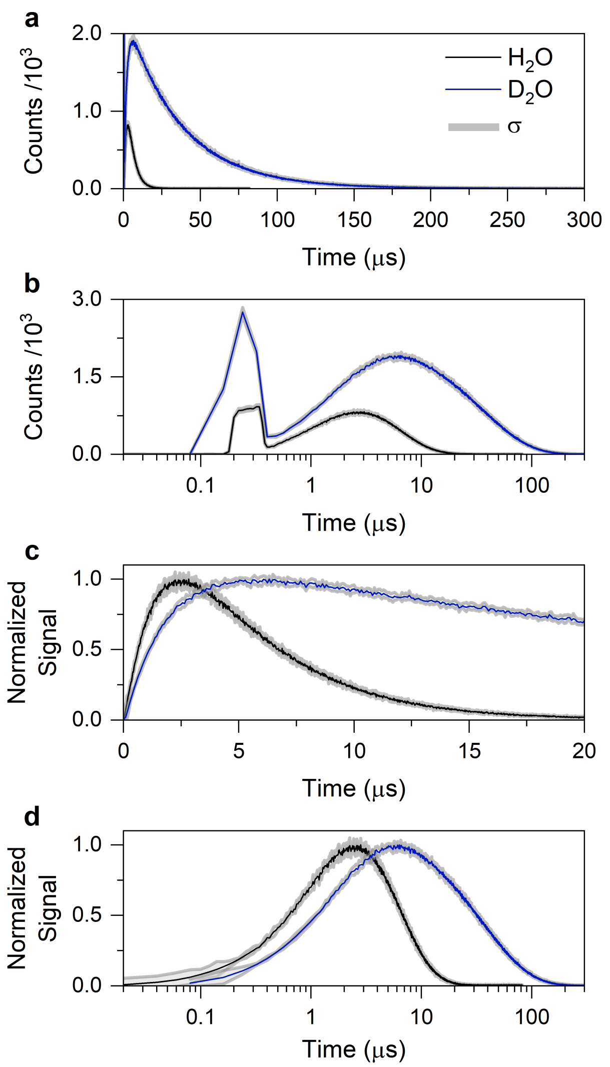

Figure S2. Comparison of $^1O_2$ phosphorescence in H_2O and D_2O. In water, the signal returns to baseline within ~50 μs owing to the short $^1O_2$ lifetime (τ_Δ = 3.5 μs), whereas in D_2O the observation window must be extended (τ_Δ = 66 ± 2 μs). (a) on a linear time scale, where the initial baseline (IBL) and steady-state tail amplitude (STA) are obscured. b) Emission on a logarithmic time scale, allowing visualization of IBL and STA. (c) Magnified view of the first 20 μs of decay, and (d) corresponding plot with time on a logarithmic scale.

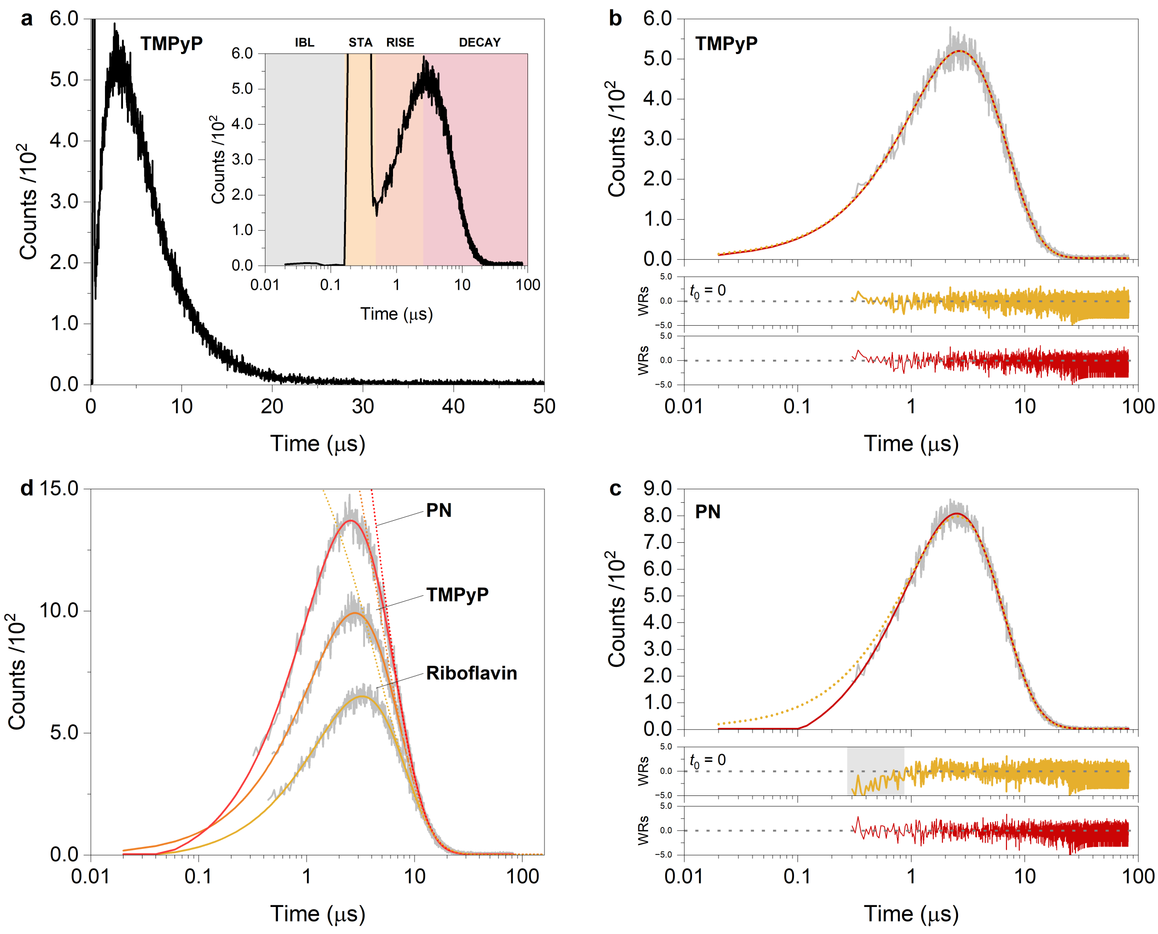

Figure S3. Kinetic traces of $^1O_2$ ($^1\Delta_g$) phosphorescence and corresponding model fits in air-saturated water, illustrating data presentation, scaling, and fitting approaches. (a) Raw experimental for TMPyP excited at 400 nm with a pulsed LED; the inset shows the same data on a $\log_{10}$ time scale to better visualize and distinguish each phase of the temporal profile. Processed kinetic trace of (b) TMPyP and (c) PN with the IBL and STA region removed and the time origin (t = 0) defined as the onset of the excitation pulse. Fits using Eq. (1) are shown with t_0 fixed at zero (orange) or included as a fitting parameter (red), along with corresponding weighted residuals. The gray area shows the region of concern. (d) Comparison of fits obtained with Eq. (1) (solid line) and a single-exponential function (tail fit, dotted line) using kinetic traces of riboflavin, TMPyP and PN produced by photoexcitation at 450 nm.

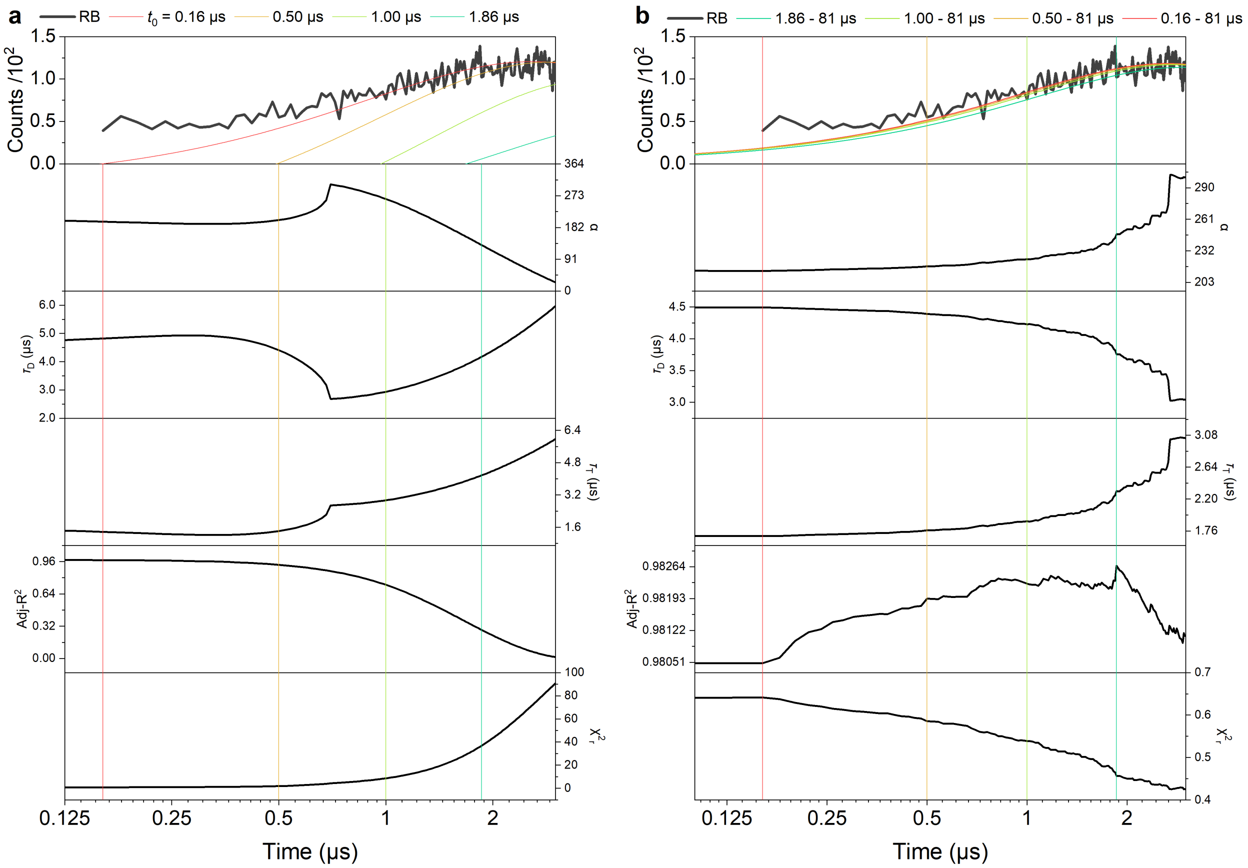

Figure S4. Effect of (a) t_0 and (b) fitting window on values of α, τ_Δ, τ_T, Adj-R^2, and χ^2_r.

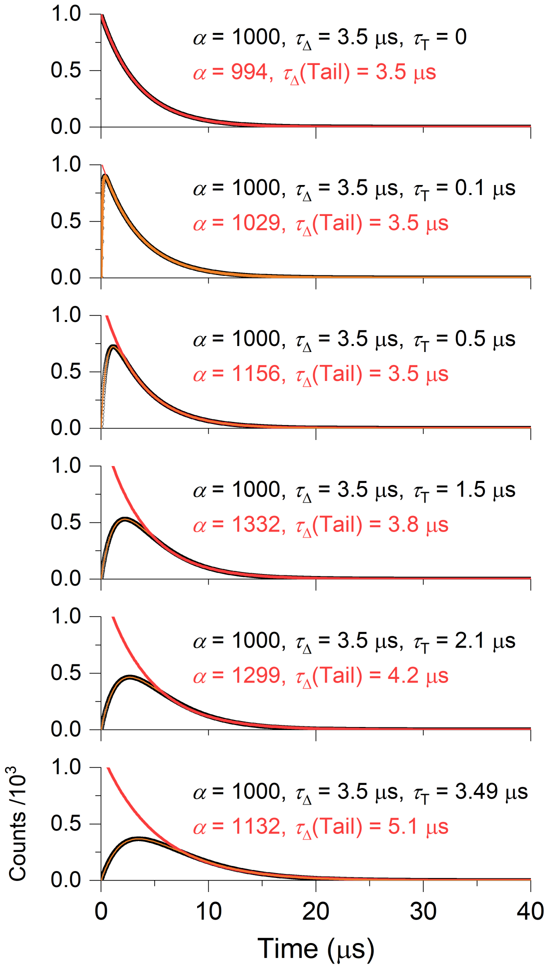

Figure S5. Effect of τ_T on the accuracy of tail fitting using an exponential function (from t = 7 μs). Simulated datasets generated using Eq. (1); the parameters used to generate the curves are shown in black, $t_0 = y_0 = 0$, $t = 0$ to 80 μs (shown until 40 μs), 4096 points. The value of τ_T increases from top to bottom. Solid red lines represent the exponential tail fits obtained with a single-exponential function; corresponding fitted parameters are shown in red for comparison. The initial signal intensity depends on the magnitude of τ_T. In the regime where the $^3PS^*$ lifetime (τ_T) ≪ τ_Δ, no exponential rise interval is observed, allowing reliable determination of τ_Δ and the amplitude parameter α by fitting with a simple exponential decay.

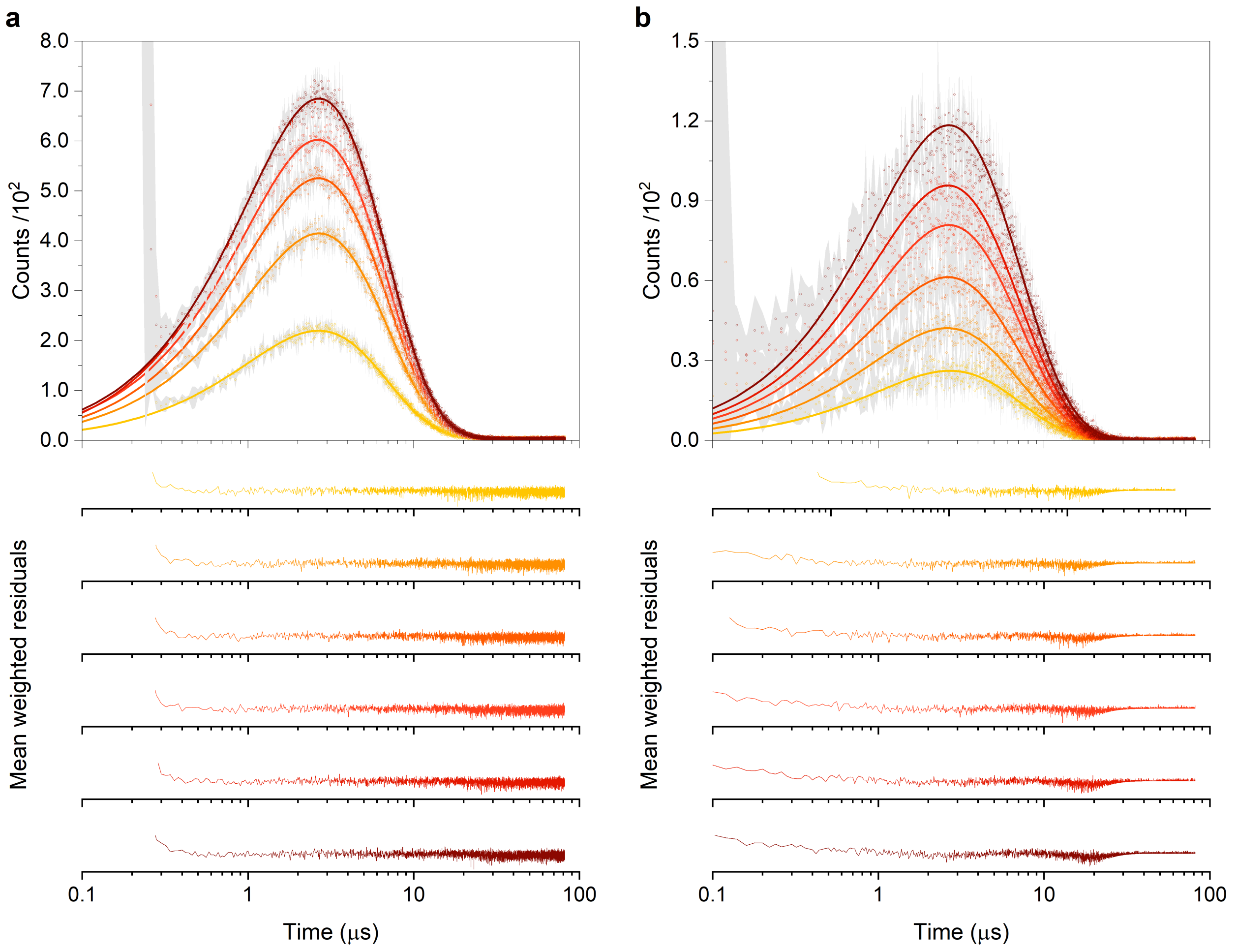

Figure S6. Effect of absorbance and excitation power on the amplitude parameter α and singlet-oxygen emission spectra. (a) Dependence of α on the absorbance of TMPyP in air-saturated H_2O; 400 nm. Solid lines are the fits to Eq. (1); gray shading indicates 1σ ($N = 7$). Dependence of α on excitation power for (b) Rose Bengal; red lines are through-origin regressions, gray shading indicates 1 σ ($N = 3$). Residuals in the STA region were intentionally left uncropped to highlight discrepancies at this limit.

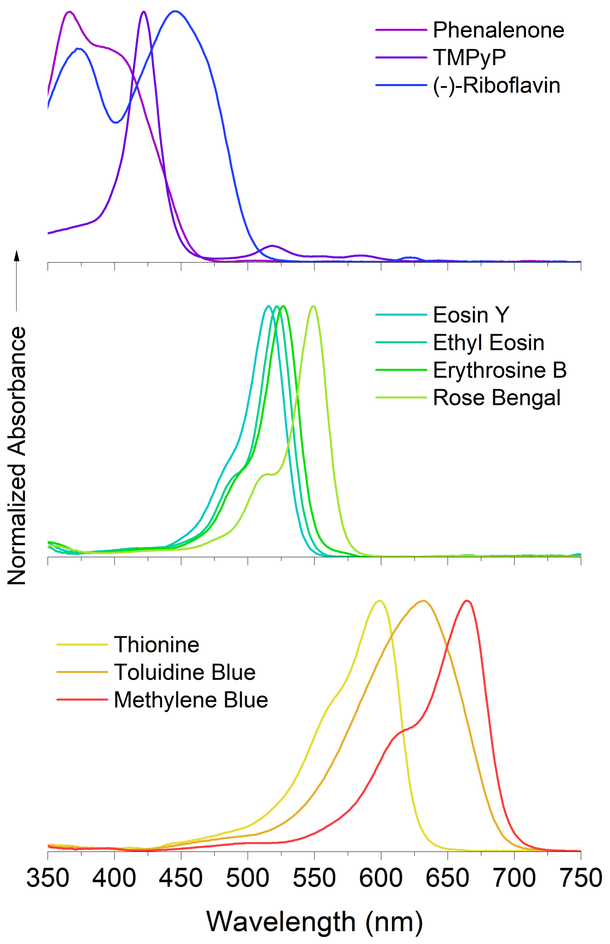

Figure S7. Normalized absorption spectra of common PSs in water. Instructions for the acquisition of UV-Vis spectra and comprehensive list of water-soluble PS and their photophysical and photochemical parameters of interest, including their Φ_{Δ}, are given in **Table S1**.

PHOTOPHYSICAL PROPERTIES OF WATER-SOLUBLE PHOTOSENSITIZERS

Table S1. Photophysical properties of common water-soluble photosensitizers.[a]

Photosensitizer	CAS#	λ^{Abs}	$\varepsilon(\lambda^{Abs})$	λ_{EX}	E_T	Ref	Φ_T	Ref	τ_T	Ref	Φ_Δ	Ref	τ_Δ	Ref	Comments
Methylene Blue	61-73-4	664	67000		33.0	3	0.52	4			0.52	5	2	6	
				650		7	0.55	7	77 ± 5	7					H_2O
									115 ± 10						D_2O
Thionine	581-64-6	600	55200	630		8			16 ± 2*	9	0.58	5			*low temperature and outgassed solution
					39.2	76	0.55	76							
Toluidine blue	92-31-9	623	39515			10							63 ± 1	11	
				630		12			2.20*	12	0.18*	12			*Deuterated phosphate buffer (PB) pH 7
					36.0	79									
Rose Bengal	632-69-9	549	95000	532	42.0	13	0.78*	14	2.2 ± 0.2	15	0.75	35	4.0 ± 0.2	15	*AOT micelle
							0.78**	16							**0.1mM NaOH
									2.1 ± 0.2	17			3.8 ± 0.3	17	
Eosin Y	548-26-5	515	112000	532	47.0	18	0.78*	70	2.1 ± 0.2**	36	0.57	15	4.0 ± 0.2	15	*isooctane + 0.1 M AOT + D_2O **PBS, pH 7.4
							0.8	19							
Rhodamine 6G	989-38-8	526	24000*	532		20	0.002	21	2.0	22	0.10	23		58	*(488nm)
					42.9	90									
Eosin	17372-87-1	630	4800	532		24	0.76 ± 0.08	24	95	94	0.57	5			
					42.1	93									
Eosin blue	18472-87-2	538	83000*			25					0.65	26			*PB, pH7.4
					39.6**	27	0.75**	27							**EtOH

Erythrosine B	15905-32-5	530	75000			28	1.07 ± 0.13*	29	2.8	30	0.68**	30			*H_2O, pH 9; **D_2O
		532*	96600*			31									*H_2O, pH 9
					44	32									
				> 455*							0.63	33			*Halogenated tungsten lamp
Riboflavin	83-88-5	373<br>445	10600<br>12500			34	0.60	35	3.0*	35	0.60	35	3.6	35	*air saturated PB pH 6.8
					50.0*	36									*EtOH/MeOH 9:1
				308<br>370		37					0.61<br>0.58	37			
Phenalenone	548-39-0	360<br>382	11250*<br>9630*		52.6	38	1.0*	38	35*	38					*MeOH
				436		39					0.98 ± 0.08	39			
					44.4**	40									**EtOH
TMPyP	36951-72-1	424	226000			41							3.68	42	
				530	33.2	43	0.92	43	165	43					
				532		44	0.65*<br>0.82**	44							*H_2O<br>**D_2O
				437		15			2.0 ± 0.2	15	0.74	15	4.1 ± 0.2	15	
Hematoporphyrin IX dihydrochloride	17696-69-4	394	137000			45									* PB, pH 7.4
				532		46	0.63*<br>0.78**	46			0.32*<br>0.53**	46			* PB, pH 7.4<br>** 2% Triton X-100
									83***	47					***deareated PB, pH 7.4
Sodium Talaporfin (NPe6)	220201-34-3	400<br>654	180000<br>40000*	355		48	0.64	48	300**	48	0.77	48	52**	48	*100 mM PB pH 7.4, 0.22 mM O_2.<br>**under argon
TPPS4	35218-75-8	434; 645				49	0.78*	49	160*<br>90**	49	0.62*	49	60 ps**	49	*Monomers, pH 7.0<br>**J aggregate

		413<br>515	370000<br>13000			50									pH 7.0
				530	33.2	43	0.78	43	420	43					
				423		15			1.9 ± 0.2	15			3.7 ± 0.2	15	
Fluorescein	2321-07-5	486	17000	510		51	0.032 ±<br>0.005*	24			0.066 ±<br>0.001	52	4.2**<br>55***	53	*0.01 mol L^{-1} NaOH<br>**H_2O<br>***D_2O
					47.2	36									EtOH/MeOH 9:1
Pterin	2236-60-4	270<br>340	11800<br>5600			54	0.23	55	3.4 ± 0.5	56					
				367	56.5	57					0.18 ± 0.02*<br>0.30 ± 0.02**	58			*pD 5.5<br>**pD 10.5
$AlPcS_4$	144082-45-1	676	200000*			59					0.38*	59			*In PBS pH 7.4 + 1 %v Triton X-100
				675		15			1.9 ± 0.2	15	0.22	15	3.7 ± 0.2	15	
							0.4*	60	750*	60					*in solution with human serum albumin

[a] $\boldsymbol{\lambda}^{\mathbf{Abs}}$ (wavelength of maximum absorption, nm), $\boldsymbol{\varepsilon}(\boldsymbol{\lambda}^{\mathbf{Abs}})$ (molar absorption coefficient at λ^{Abs}, L mol^{-1} cm^{-1}), $\boldsymbol{\lambda}_{\mathbf{EX}}$ (excitation wavelength, nm), $\boldsymbol{E}_{\mathbf{T}}$ (triplet-state energy, kcal mol^{-1}), $\boldsymbol{\Phi}_{\mathbf{T}}$ (triplet quantum yield), $\boldsymbol{\tau}_{\mathbf{T}}$ (triplet-state lifetime, µs), $\boldsymbol{\Phi}_{\boldsymbol{\Delta}}$ (singlet oxygen quantum yield), and $\boldsymbol{\tau}_{\boldsymbol{\Delta}}$ (singlet oxygen lifetime, µs); **Ref** (reference).

NOTES

Note S1 – A Primer on Singlet Oxygen

Molecular oxygen ($^3O_2$), introduced into the biosphere by oxygenic photosynthesis, became the dominant oxidant enabling aerobic life.[61] In its triplet ground-state ($^3\Sigma_g^-$), direct oxidation of singlet organic substrates is spin-forbidden, imparting remarkable kinetic stability to organic matter[62] and preventing spontaneous combustion under ambient conditions.[63] Upon electronic excitation, however, oxygen can populate singlet excited states with very different reactivity. The lower-energy $^1\Delta_g$ state lies ~0.98 eV (~1270 nm) above the ground state and exhibits lifetimes of ~70 µs in nonpolar solvents and <1 µs in the presence of chemical quenchers such as those encountered in biological environments.[64] Spectral data for the $O_2(^1\Delta_g) \rightarrow O_2(^3\Sigma_g^-)$ transition are 7849 ± 3 cm^{-1} (1273.8 ± 0.5 nm) in water and 7850 ± 1 cm^{-1} (1273.7 ± 0.2 nm) in D_2O.[64, 65] The higher energy $^1\Sigma_g^+$ state lies ~1.63 eV above the ground state and decays rapidly (<1 ns) to the $^1\Delta_g$ state. Hence, the term *singlet oxygen* ($^1O_2$) refers almost exclusively to the $^1\Delta_g$ state, which is the most relevant state to photochemical reactivity and photobiological processes. Owing to its electrophilic character and spin compatibility with singlet substrates, $^1O_2$ reacts readily with organic molecules and serves as a key intermediate in photochemical and photobiological transformations, as well as a versatile reagent in synthetic organic chemistry, used for cycloadditions, *ene* reactions, and selective oxidations.[66, 67]

Singlet oxygen is highly sensitive to collisional quenching. In the gas phase, an isolated molecule exhibits a radiative half-life of ~45 min, which decreases to ~9 min at 1 atm.[68-70] Because the $^1O_2\,(^1\Delta_g) \rightarrow {}^3O_2(^3\Sigma_g^-)$ transition is spin- ($\Delta S \neq 0$) and symmetry-forbidden ($g \nleftrightarrow g$, $\Delta\Lambda = 2$), deactivation is dominated by phosphorescence. In solution, τ_Δ varies strongly with the solvent:

3.5 μs in H_2O, 67 μs in D_2O, and up to 70 ms in CCl_4.[64, 71-73] These differences arise from efficient nonradiative deactivation mechanisms, including electronic-to-vibrational energy transfer, charge-transfer quenching, and electronic energy transfer to suitable acceptors.[73, 74] Solvents also influence the electronic structure of O_2, enhancing intersystem crossing by breaking molecular symmetry and increasing spin-orbit coupling.[70, 74, 75] The pronounced kinetic isotope effect ($k^H/k^D \approx 20$) observed when comparing $^1O_2$ deactivation in H_2O and D_2O supports this mechanism and has been attributed to quantum tunneling differences between $^1(O_2\cdots H_2O)$ and $^1(O_2\cdots D_2O)$ complexes.[75, 76]

Note S2 – Models for Kinetic Analysis

Homogeneous Systems

A comprehensive treatment of the kinetics and dynamics of singlet oxygen generation and deactivation is provided in the seminal works of Wilkinson and coauthors,[77, 78] as well as in subsequent studies.[70, 71, 74, 79, 80] Here, our goal is to present an operational description of the relevant variables under the following simplifying conditions: (*i*) the PS remains intact during the process,[81] (*ii*) no external quenchers other than $^3O_2$ are present, (*iii*) interactions between oxygen and the $^1PS^*$ are negligible, and (*iv*) the concentration of $^3O_2$ is constant.

The photosensitized formation of $^1O_2$ can be described through the following processes:

• Photoexcitation of the PS and deactivation of $^1PS^*$

$PS + h\nu \rightarrow {}^1PS^*$	Rate I_{abs} in Einstein s^{-1}
${}^1PS^* \rightarrow PS + h\nu_F$	Fluorescence, k_F, s^{-1}
${}^1PS^* \rightarrow PS + heat$	Non-radiative decay of $^1PS^*$, $^1k_{nr}$, s^{-1}
${}^1PS^* \rightarrow {}^3PS^*$	Intersystem Crossing, k_{ISC}, s^{-1}

• **Decay of $^3PS^*$**

$^3PS^* \rightarrow PS + h\nu_P$ — PS phosphorescence, k_P, s^{-1}

$^3PS^* \rightarrow PS + \text{heat}$ — Non-radiative decay of $^3PS^*$, $^3k_{ns}$, s^{-1}

• **Quenching of $^3PS^*$ by molecular oxygen**

$^3PS^* + {^3O_2} \rightleftharpoons {^3[PS\cdots O_2]^*}$ — Triplet collision complex formation, $3k_{on}/k_{off}$

$^3PS^* + {^3O_2} \rightleftharpoons {^5[PS\cdots O_2]^*}$ — Quintuplet collision complex formation, $5k_{on}/k_{off}$

$^3PS^* + {^3O_2} \rightleftharpoons {^1[PS\cdots O_2]^*}$ — Singlet collision complex formation, k_{on}/k_{off}

From these collision complexes:

$^1[PS\cdots O_2]^* \rightarrow PS + {^1O_2}$ — Singlet oxygen formation

$^3[PS\cdots O_2]^* \rightarrow PS + {^3O_2}$ — $^3PS^*$ quenching

These processes are often simplified to the following bimolecular reactions:

$^3PS^* + {^3O_2} \rightarrow PS + {^1O_2}$ — Bimolecular singlet oxygen formation, k_Δ

$^3PS^* + {^3O_2} \rightarrow PS + {^3O_2}$ — Bimolecular quenching, k_d

• **Decay of singlet oxygen**

$^1O_2 \rightarrow {^3O_2} + h\nu_\Delta$ — $^1O_2$ phosphorescence at ~1270 nm, $^\Delta k_r$, s^{-1}

$^1O_2 \rightarrow {^3O_2}$ — Singlet oxygen decay, $^\Delta k_{nr}$, s^{-1}

In a homogeneous environment, the lifetime of the singlet excited PS (τ_S) is:

$$\tau_S = \frac{1}{k_S} = \frac{1}{k_F + {^1k_{nr}} + k_{ISC}} \tag{1}$$

The lifetime of the triplet excited PS (τ_T) is:

$$\tau_T = \frac{1}{k_T} = \frac{1}{k_P + {^3k_{nr}} + (k_\Delta + k_d)[^3O_2]} \tag{2}$$

The lifetime of $^1O_2$ is:

$$\tau_\Delta = \frac{1}{{}^\Delta k_\mathrm{r} + {}^\Delta k_\mathrm{nr}} \tag{3}$$

All excited state concentrations are understood to be time-dependent, although the explicit notation (t) is omitted for clarity. Subscript '0' denotes the initial concentration at $t = 0$. Under these conventions, and considering that $[{}^1PS^*]_0 = [{}^3PS^*]_0 = [{}^1O_2]_0 = 0$ with $[{}^3O_2]$ assumed constant, the population dynamics following an instantaneous excitation pulse can be described as follows:

$$\frac{d[{}^1\mathrm{PS}^*]}{dt} = -(k_F + {}^1k_\mathrm{nr} + k_\mathrm{ISC})[{}^1\mathrm{PS}^*] \tag{4}$$

$$\frac{d[{}^3\mathrm{PS}^*]}{dt} = k_\mathrm{ISC}[{}^1\mathrm{PS}^*] - \frac{1}{\tau_\mathrm{T}}[{}^3\mathrm{PS}^*] \tag{5}$$

$$\frac{d[{}^1\mathrm{O}_2]}{dt} = k_\Delta[{}^3\mathrm{O}_2][{}^3\mathrm{PS}^*] - \frac{1}{\tau_\Delta}[{}^1\mathrm{O}_2] \tag{6}$$

From Eq. (4),

$$[{}^1\mathrm{PS}^*] = [{}^1\mathrm{PS}^*]_0\, e^{-t/\tau_\mathrm{S}} \tag{7}$$

Solving Eq. (5) with Eq. (7):

$$[{}^3\mathrm{PS}^*] = [{}^1\mathrm{PS}^*]_0 \frac{k_\mathrm{ISC}}{(1/\tau_\mathrm{T}) - (1/\tau_\mathrm{S})}\left(e^{-t/\tau_\mathrm{T}} - e^{-t/\tau_\mathrm{S}}\right) \tag{8}$$

The transient concentration of $^1O_2$ is obtained by solving Eq. (6) with Eq. (8):

$$\begin{aligned}[{}^1\mathrm{O}_2] = [{}^1\mathrm{PS}^*]_0 &\frac{k_\mathrm{ISC}}{k_\mathrm{F} + {}^1k_\mathrm{nr} + k_\mathrm{ISC}} \frac{k_\Delta[{}^3\mathrm{O}_2]}{k_\mathrm{P} + {}^3k_\mathrm{nr} + (k_\Delta + k_\mathrm{d})[{}^3\mathrm{O}_2]} \\ &\times \left[\frac{\tau_\Delta}{\tau_\Delta - \tau_\mathrm{T}}\left(e^{-t/\tau_\Delta} - e^{-t/\tau_\mathrm{T}}\right) - \frac{\tau_\Delta}{\tau_\Delta - \tau_\mathrm{S}}\left(e^{-t/\tau_\Delta} - e^{-t/\tau_\mathrm{S}}\right)\right]\end{aligned} \tag{9}$$

The number of $^1O_2$ molecules produced per photon absorbed by the PS is the singlet oxygen quantum yield (Φ_Δ):[81]

$$\Phi_\Delta = \frac{\text{number of } {}^1\mathrm{O}_2 \text{ molecules generated}}{\text{number of photons absorbed}} \tag{10}$$

Because photosensitized $^1O_2$ generation is a multistep process, the quantum yield is a product of efficiencies[82,83] and can be determined by considering the rate constants leading to $^1O_2$ divided by the sum of the rate constants of all competing processes. Neglecting $^1O_2$ formation from quenching of $^1PS^*$ by $^3O_2$, we have:

$$\Phi_\Delta = \eta_\mathrm{T}\,\eta_\mathrm{ET} = \frac{k_\mathrm{ISC}}{k_\mathrm{F} + {}^1k_\mathrm{nr} + k_\mathrm{ISC}}\,\frac{k_\Delta[^3\mathrm{O}_2]}{k_\mathrm{P} + {}^3k_\mathrm{nr} + (k_\Delta + k_\mathrm{d})[^3\mathrm{O}_2]} \tag{11}$$

where η_T and η_ET are the efficiencies of triplet formation and energy transfer to produce $^1O_2$, respectively.

If $\tau_\mathrm{S} \ll \tau_\mathrm{T}$ and τ_Δ, Eq. (9) reduces to:

$$[^1\mathrm{O}_2] = [^1\mathrm{PS}^*]_0\,\Phi_\Delta\,\frac{\tau_\Delta}{\tau_\Delta - \tau_\mathrm{T}}\left(e^{-t/\tau_\Delta} - e^{-t/\tau_\mathrm{T}}\right) \tag{12}$$

Experimentally, the phosphorescence signal of $^1O_2$ at ~1270 nm, S, is directly proportional to $[^1O_2]$:

$$S = \frac{\kappa}{n_\mathrm{r}^2}\,{}^\Delta k_\mathrm{r}\,[^1\mathrm{O}_2] \tag{13}$$

where κ refers to geometrical and electronic factors of the system, n_r is the reactive index of the medium at the excitation wavelength, and $^\Delta k_\mathrm{r}$ is the rate constant for the radiative decay of $^1O_2$. Substituting Eq. (12) in Eq. (13):

$$S = \alpha\,\frac{\tau_\Delta}{\tau_\Delta - \tau_\mathrm{T}}\left(e^{-t/\tau_\Delta} - e^{-t/\tau_\mathrm{T}}\right) \tag{14}$$

where,

$$\alpha = \frac{\kappa}{n_\mathrm{r}^2}\,{}^\Delta k_\mathrm{r}\,[^1\mathrm{PS}^*]_0\,\Phi_\Delta \tag{15}$$

This expression follows the canonical form introduced by Nonell and Braslavsky,[84] building on the considerations of Wilkinson and coauthors,[77] and later modified or extended.[15, 71, 80, 85-89]

From Eq. (15):

$$\Phi_\Delta = \alpha \frac{n_r^2}{\kappa\ {}^\Delta k_r\ [{}^1\mathrm{PS}^*]_0} \tag{16}$$

Assuming low saturation and low fluence (where ground-state depletion is small and multi-photon effects are negligible), the average concentration of singlet-excited PS, $[{}^1PS^*]_0$, created in the irradiated volume *V*, is defined as:

$$[{}^1\mathrm{PS}^*]_0 = \frac{E_{\mathrm{flash}}\,\lambda}{h\,c}(1 - 10^{-A(\lambda)})\,\frac{\Phi_\mathrm{S}}{N_\mathrm{A}\,V} \tag{17}$$

where E_{flash} is the energy of the excitation pulse, *h* is Planck's constant, *c* is the speed of light in vacuum, λ is the excitation wavelength, N_A is Avogadro's constant and Φ_S is the quantum yield for forming $^1PS^*$ from an absorbed photon (often equal 1 for initial singlet creation). For very large E_{flash} or very high $A(\lambda)$, corrections may be needed.

The relative determination of Φ_Δ for an unknown sample is based on the general comparison with the singlet oxygen quantum yield of a standard, $\Phi_\Delta{}^{Std}$:

$$\Phi_\Delta = \Phi_\Delta^{\mathrm{Std}}\,\frac{\alpha}{\alpha^{\mathrm{Std}}}\,\frac{[{}^1\mathrm{PS}^*]_0^{\mathrm{Std}}}{[{}^1\mathrm{PS}^*]_0}\,\frac{n_r^2}{(n_r^{\mathrm{Std}})^2}\,\frac{{}^\Delta k_r^{\mathrm{Std}}}{{}^\Delta k_r} \tag{18}$$

Substituting Eq. (17) in Eq. (18):

$$\Phi_\Delta = \Phi_\Delta^{\mathrm{Std}}\,\frac{\alpha}{\alpha^{\mathrm{Std}}}\,\frac{1 - 10^{-A(\lambda)^{\mathrm{Std}}}}{1 - 10^{-A(\lambda)}}\,\frac{E_{\mathrm{flash}}^{\mathrm{Std}}}{E_{\mathrm{flash}}}\,\frac{\lambda^{\mathrm{Std}}}{\lambda}\,\frac{\Phi_\mathrm{S}^{\mathrm{Std}}}{\Phi_\mathrm{S}}\,\frac{V}{V^{\mathrm{Std}}}\,\frac{n_r^2}{(n_r^{\mathrm{Std}})^2}\,\frac{{}^\Delta k_r^{\mathrm{Std}}}{{}^\Delta k_r} \tag{19}$$

which under identical experimental conditions for both unknown sample and standard simplify to:

$$\Phi_\Delta = \Phi_\Delta^{\mathrm{Std}}\,\frac{\alpha}{\alpha^{\mathrm{Std}}} \tag{20}$$

Most importantly, the radiative rate constant of $^1O_2$ is highly sensitive to even small variations in the solvent refractive index.[90] Therefore, the presence of the n_r term in previous equations results from rigorous derivation and should not be interpreted as a means of facilitating comparisons of data obtained in different solvents.

In the determination of the Φ_Δ, the use of α was preferred over integration of the full emission decay. In aqueous systems, the τ_Δ is very short and largely governed by nonradiative quenching processes, which causes the integrated emission area to be highly sensitive to baseline stability, detector noise, and the efficiency of STA removal. Small variations in baseline correction or truncation of early-time data can therefore introduce significant uncertainty into the integrated signal. In contrast, α obtained from exponential fitting of the decay, is directly proportional to the number of absorbed photons and is less affected by baseline drift or late-time noise, providing a more robust and reproducible metric for relative Φ_Δ determination under these conditions.

The Diffusion–Reaction Model

In a heterogeneous environment, the spatial and temporal evolution of $^1O_2$ must account for both generation and diffusion processes within regions of distinct physicochemical properties.[2, 91, 92] A spherical single bilayer vesicle of diameter d is represented by n concentric spherical shells of uniform thickness Δx = 1 nm, indexed j = 1, ..., n from the center. The radial coordinate of shell j is $r_j = j\Delta x$. For d = 78 nm, shells j = 35...39 represent a 4 nm lipid bilayer; all other shells are water (interior and exterior). Each shell has volume $V_j = 4\pi\, r_j^2\, \Delta x$ and an interface with shell j + 1 of area

$$A_{j,j+1} = 4\pi(\Delta x)^2\, j(j+1) \tag{21}$$

The time-dependent concentration of $^1O_2$ in shell j at time t [$c_j(t)$], obeys the radial diffusion–reaction equation derived from Fick's laws:

$$\frac{\partial c_j}{\partial t} = D_j\left[\frac{1}{r_j^2}\frac{\partial}{\partial r_j}\left(r_j^2\frac{\partial c_j}{\partial r_j}\right)\right] - \frac{c_j}{\tau_j} + R_j(t), \qquad r_j = j\,\Delta r \tag{22}$$

where D_j is the diffusion coefficient in shell j (distinct for lipid and aqueous regions), τ_j is the local $^1O_2$ lifetime, R_j(t) is the source term due to $^3PS^*$ decay, and $r_j = j\Delta r$ is the radial coordinate.

Singlet oxygen is generated only in layers containing the PS. The source function follows the PS triplet decay kinetics:

$$R_j(t) = \eta_{\mathrm{ET}}\,[^3\mathrm{PS}^*]_0\,\frac{e^{-t/\tau_T}}{\tau_T}\,\delta_{j,j_{\mathrm{PS}}} \tag{23}$$

where τ_T is the $^3PS^*$ lifetime and η_{ET} is the efficiency of energy transfer to O_2.

For the discrete spherical shell model, Eq. (21) is expressed in finite-difference form as:

$$\Delta c_j = \frac{D_j A_{j,j+1}}{V_j\,\Delta r}\,(c_{j+1} - c_j) - \frac{D_{j-1} A_{j-1,j}}{V_j\,\Delta r}\,(c_j - c_{j-1}) - \frac{c_j\,\Delta t}{\tau_j} + R_j\,\Delta t \tag{24}$$

where, $A_{j,j+1} = 4\pi(\Delta r)^2\, j(j+1)$ is the interfacial area, and $V_j = 4\pi r_j^2 \Delta r$ is the shell volume. The local time step Δt was chosen as 0.125 ns to ensure numerical stability.

At phase boundaries (lipid/water), diffusion is calculated using the diffusion coefficient of the lipid phase, as it is the slower of the two. Solubility differences are incorporated as partition factors. If the solubility of $^1O_2$ in lipid is S times higher than in water, the interfacial concentration gradient is corrected as:

$$\left(\frac{\Delta c}{\Delta r}\right)_{\mathrm{interface}} = \frac{c_{j+1} - c_j/S}{\Delta r} \tag{25}$$

At each time step, the total populations of $^1O_2$ in the lipid bilayer located at layers 35 to 39 and in water (layers 1 to 34 and from 40 on) are evaluated as:

$$n_L(t_i) = \sum_{j=35}^{39} c_j(t_i)\,V_j, \qquad n_W(t_i) = \sum_{j=1}^{34} c_j(t_i)\,V_j + \sum_{j=40}^{n} c_j(t_i)\,V_j \tag{26}$$

The detected $^1O_2$ phosphorescence signal at ~1270 nm corresponds to the weighted sum of these populations:

$$S(t_i) = A\,n_L(t_i) + B\,n_W(t_i) + C \tag{27}$$

where A and B are proportional to the local radiative rate constants k_r^{L} and k_r^{W} in the lipid and aqueous phases, respectively, and C accounts for time-independent background counts. The amplitude ratio A/B therefore reflects the ratio of radiative rate constants:

$$\frac{A}{B} = \frac{k_r^{(L)}}{k_r^{(W)}} \tag{28}$$

Experimental kinetics are fitted by minimizing the reduced χ^2 function:

$$\chi^2_{\text{red}} = \frac{1}{N-n}\sum_i \left[\frac{S_{\exp}(t_i) - S(t_i)}{\sigma_i}\right]^2 \tag{29}$$

where, N is the number of channels and n is the number of fit parameters. The optimal parameter set (τ_T, $\tau_{\Delta,W}$, A/B) defines the most probable combination of PS triplet lifetime, singlet oxygen lifetime in water, and radiative rate ratio between phases. Channel uncertainties are defined by σ_i.

Note S3 – Challenges in Non-Linear Curve Fitting

Degeneracies are frequently observed in the analysis of rise-and-decay kinetics, where different parameter combinations can produce equally valid fits.[93, 94] In particular, the rise and decay constants can be interchanged without changing the overall fit, requiring one's attention to the context for proper interpretation. To illustrate this problem, consider the empirical biexponential (difference) mathematical function:

$$I(t) = A \cdot \left(e^{-t/\tau_1} - e^{-t/\tau_2}\right) \tag{30}$$

that can be used to fit kinetics that exhibit a rise followed by a decay. This function is antisymmetric under exchange of the time constants: swapping τ_1 and τ_2 is equivalent to changing the sign of the amplitude A. Consequently, the parameter indexed as τ_1 does not uniquely correspond to the rising phase (the same applies to the associated rate constant k_1). To avoid

ambiguity, the convention $\tau_1 < \tau_2$ is often adopted, with the understanding that the kinetic interpretation arises from their interplay rather than from either constant individually.

In the context of chemical reactions, consider the consecutive irreversible first-order steps:

$$\mathrm{R} \xrightarrow{k_1} \mathrm{I} \xrightarrow{k_2} \mathrm{P}$$

With $c_{\mathrm{R},0}$ as the concentration of R at $t = 0$ and $c_{\mathrm{I},0} = 0$, the exact solutions are:

$$c_{\mathrm{R}}(t) = c_{\mathrm{R},0}\, e^{-t/\tau_1} \tag{31}$$

$$c_{\mathrm{I}}(t) = \frac{c_{\mathrm{R},0}\ \tau_2}{\tau_1 - \tau_2}\left(e^{-t/\tau_1} - e^{-t/\tau_2}\right) \tag{32}$$

which is equivalent to the empirical form in Eq. (30). As reaction proceeds, the slower step controls the tail:

$$c_{\mathrm{I}}(t) \sim \begin{cases} \dfrac{c_{\mathrm{R},0}\ \tau_2}{\tau_1 - \tau_2}\, e^{-t/\tau_1}, & \tau_1 > \tau_2 \\[2ex] -\dfrac{c_{\mathrm{R},0}\ \tau_2}{\tau_1 - \tau_2}\, e^{-t/\tau_2}, & \tau_2 > \tau_1 \end{cases} \tag{33}$$

Hence, again the tail is governed by whichever τ is larger, not by a fixed index, and neither indexed lifetime inherently denotes the rise step. In unconstrained nonlinear fitting, solutions that interchange τ_1 and τ_2 (with a compensating change in A) are therefore common, especially in automated analyses across multiple datasets.

This degeneracy is reflected not only in the algebraic symmetry of the biexponential but also in the statistical landscape of the fit. A related statistical perspective is that the χ^2 surface in (τ_{T}, τ_{Δ}) space often exhibits extended valleys or "hyperplanes" of nearly constant χ^2 (**Figure S8**). In practice, this means that different combinations of rise and decay constants yield statistically indistinguishable fits, particularly at modest signal-to-noise ratios. The χ^2 minimum is therefore not a sharp attraction point but rather a flat ridge (e.g., along the diagonal where an increase in τ_{T} can be offset by a decrease in τ_{Δ}). Such degeneracies make nonlinear fitting sensitive to artifacts

or secondary signals in the data and can cause automated procedures to converge on different but equally valid parameter sets. To break this symmetry, it is often necessary to impose independent physical constraints (such as fixing one lifetime from an external measurement), ensuring that the fit converges to a mechanistically meaningful solution.

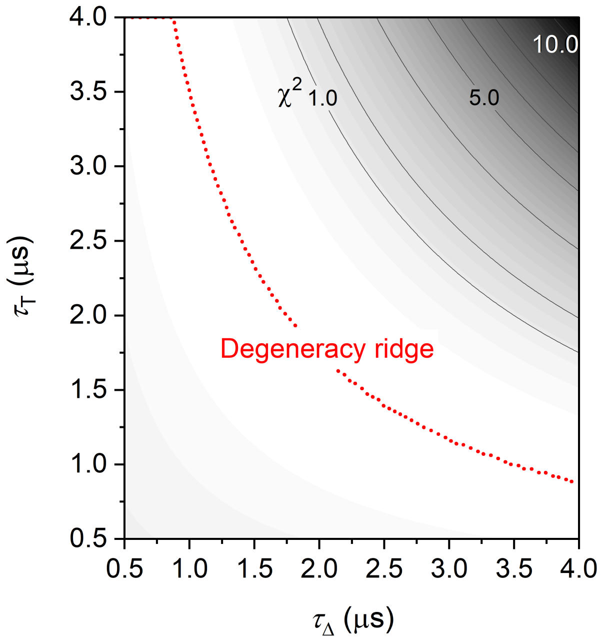

Figure S8. Contour plot of the reduced χ^2 surface in (τ_T, τ_Δ) space (here equivalent to τ_1 and τ_2), illustrating the degeneracy ridge where multiple parameter combinations yield equally good fits. Here, χ^2 was modelled as $\chi^2(\tau_T, \tau_\Delta) = [(\tau_T\ \tau_\Delta/3.5) - 1]^2$, purely to illustrate the degeneracy valley of nearly constant χ^2 along the hyperbola $\tau_T\ \tau_\Delta \approx 3.5$. The red dashed line (ridge) marks the locus of minima, emphasizing that increases in one variable can be compensated by decreases in the other, resulting in indistinguishable fits.

A practical remark is that, while the empirical and mechanistic forms [Eqs. (30) and (32)] both describe rise-and-fall kinetics analogous to those observed in $^1O_2$ formation/depletion, first-

principles identification of variables and initial/boundary conditions is of paramount importance for mechanistic interpretation; fitting alone cannot unequivocally determine rate assignments without those constraints. Similarly, fitting alone cannot reliably separate contributions from distinct $^1O_2$ environments and other emitters, as their parameters may compensate for one another. Independent physical constraints are therefore essential to achieve meaningful convergence. In the case of τ_T and τ_Δ, the former parameter should, when possible, be determined through transient absorption experiments.

Note S4 – Processing Raw Data

Baseline Correction

The treatment of baseline intensity may impact the accuracy of kinetic parameter estimation in singlet oxygen phosphorescence decay analysis, particularly affecting the amplitude parameter. Rather than manually subtracting a predetermined baseline, a y-offset parameter y_0 should be included as a fitted parameter in the exponential model. This approach prevents error propagation of baseline estimation uncertainties into the amplitude and kinetic parameters. Manual baseline subtraction followed by fitting to a baseline-free model may introduce systematic bias in $S(0)$, as this amplitude parameter becomes mathematically coupled to baseline errors and may compensate for residual offsets by adopting non-physical values. For example, if too much baseline is subtracted, the fitted amplitude artificially increases to compensate for the resulting negative offset, leading to overestimated singlet oxygen production yields. Conversely, insufficient baseline correction forces the amplitude parameter to absorb the excess signal, underestimating the true kinetic amplitude. Including y_0 as a fitted parameter ensures that $S(0)$ accurately represents the physical signal amplitude from singlet oxygen decay, while baseline

variations are appropriately absorbed into the y_0 term, preserving the physical meaning of the kinetic constants τ_Δ and τ_T and providing robust uncertainty estimates for all parameters (**Figure S9**).

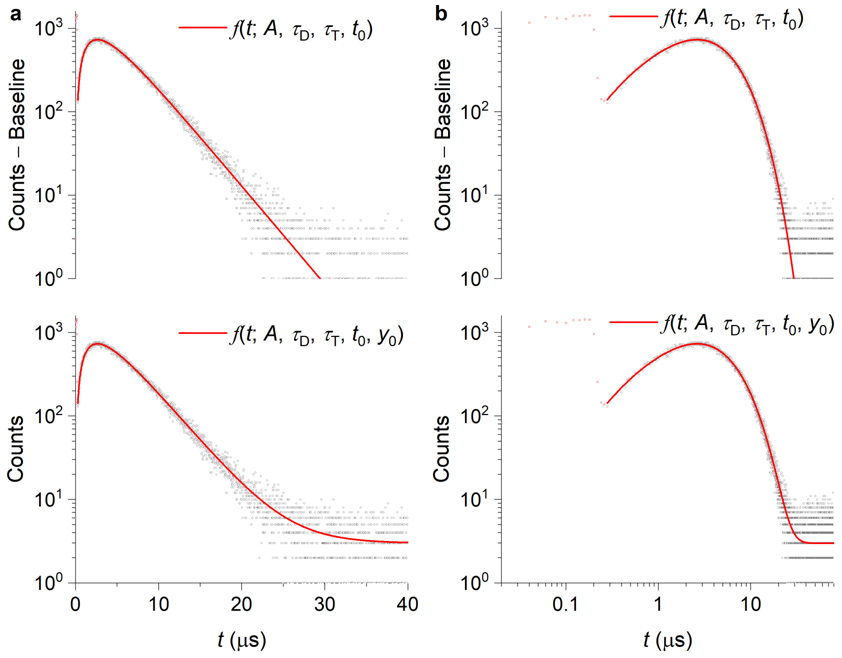

Figure S9. Effect of baseline correction on $^1O_2$ phosphorescence signal and inclusion of a y_0 offset term into the fitting model. Considering uncertainties, fitting parameters are identical: $\alpha = 1451 \pm 8$, $t_0 = 0.091 \pm 0.004$ μs, $\tau_\Delta = 3.68 \pm 0.02$ μs, $\tau_T = 1.81 \pm 0.02$ μs, $y_0 = 3.02$, $R^2 = 0.99728$. Masked points (laser artifact) are colored in red.

Short-Time Artifacts

The analysis of pre-signal artifacts is also of critical importance to validate the $^1O_2$ signal. Short-time artifacts (STAs) in time-resolved optical measurements are widely attributed to heat-producing and carrier-trapping processes inside the detector.[95] In photomultiplier tubes (PMTs), a dominant contribution to the dark count rate is thermionic emission of electrons from the photocathode/dynodes; its strong temperature dependence is well documented and approximately

follows Richardson-type behavior.[96] In semiconductor single-photon detectors (APDs/SPADs), thermally generated carriers (band-to-band or via defect states) can trigger avalanches and, together with after pulsing from trapped avalanche charges subsequently released, produce time-localized excess counts that appear as STAs.[97] In addition, some LEDs exhibit long-wavelength spectral tails beyond their nominal peak that can leak into near-IR-sensitive detection channels and elevate the short-time background, even though it is typically far below true IR-LED output.[98] Numerous reports confirm the presence of STAs across different systems and experimental setups, especially when the oxygen concentration is low, as a consequence of PS phosphorescence enhancement.[99-101] Some groups have attempted experimental subtraction using band-pass filters centered at wavelengths adjacent to the $^1O_2$ emission, but such methods are only reliable when the interfering emission is spectrally well-defined.[102, 103] Additional sources of interference include laser-related artifacts, luminescence from optical components, and sample holders. These can be minimized by careful selection of quartz-based optics and pre-testing components for background luminescence.

Note S5 – Aqueous Media

Buffer Selection

Make sure the water used to prepare the PS solutions is ultrapure by performing negative control experiments in the absence of PS. Choose buffers wisely if needed. For experiments requiring controlled pH conditions, buffers must be chosen carefully since they may affect Φ_Δ and τ_Δ through different mechanisms. For example, histidine buffer can generate new PS upon irradiation.[104] HEPES buffer prevented light-mediated enzymatic inactivation, suggesting a protective role against photosensitization under certain conditions.[105] Acetate and carbonate

buffers can catalyze the photolysis of riboflavin, increasing its degradation rate[106] MOPS buffers undergo photoinduced redox processes.[107, 108] Phosphate-buffered saline (PBS) has shown significantly lower photosensitizing activity compared to other media, indicating a stabilizing effect against photosensitization[109] Most important, phosphate anions are known to be general base catalysts, which may promote hydrolysis of unknown PSs. Nevertheless, PBS showed no effect on the values of τ_Δ and τ_T of TMPyP compared to water.[110]

Cell Culture Media and DMSO

The composition of cell culture media exerts a significant influence on photosensitization outcomes under light irradiation.[109] Commonly used media such as Dulbecco's Modified Eagle's Medium (DMEM), Roswell Park Memorial Institute 1640 (RPMI), and Minimum Essential Medium (MEM) display inherent photosensitizing properties due to riboflavin, a key component that absorbs strongly in the blue light region and promotes reactive oxygen species (ROS) generation. Phenol red, frequently included as a pH indicator, further modulates these effects by absorbing visible light and reducing transmission, thereby diminishing the apparent activity of exogenous PSs with overlapping absorption spectra. In contrast, other medium constituents, such as sodium pyruvate and fetal bovine serum (FBS), act as deactivators of hydrogen peroxide and other reactive oxygen species, thereby ameliorating phototoxicity. These findings underscore the fact that media composition can both potentiate and attenuate photosensitization, introducing confounding effects that must be carefully controlled for reliable interpretation of photobiological and photodynamic experiments.

If one is comparing absolute values of Φ_Δ and τ_Δ with samples in pure water, discretion is advised. DMSO is frequently used to increase the solubility of organic compounds in water, since it forms strong hydrogen-bonded complexes with water molecules, creating mixed solvent species

that reduce the extent of water-water interactions and provide a solvation environment capable of stabilizing both polar and nonpolar solutes.[111] However, this change in the solvent microenvironment may have a dramatic effect on the properties of both $^3PS^*$ and $^1O_2$, affecting the values of Φ_Δ and τ_Δ. The good coordination between water and DMSO shields quenching end groups (–OH) resulting in longer decay times compared to either solvent alone. Even small amounts result in changes in the [$^1O_2$] (**Figure S10**). For example, 1% v/v DMSO [~0.25 mol%, ~0.14 mol L^{-1}] reduced Φ_Δ by ~9% (p = 0.06, N = 7), which is small and bordering statistically significant. Additionally, the refractive index (n_D) and viscosity [η_V(25 ºC)] of DMSO[112] are 1.4793 (20 ºC) and 1.987 (25ºC) cP, respectively, which differ significantly from those of water (n_r = 1.333, ζ = 0.890 (25 ºC)), requiring correction for the calculation of Φ_Δ.

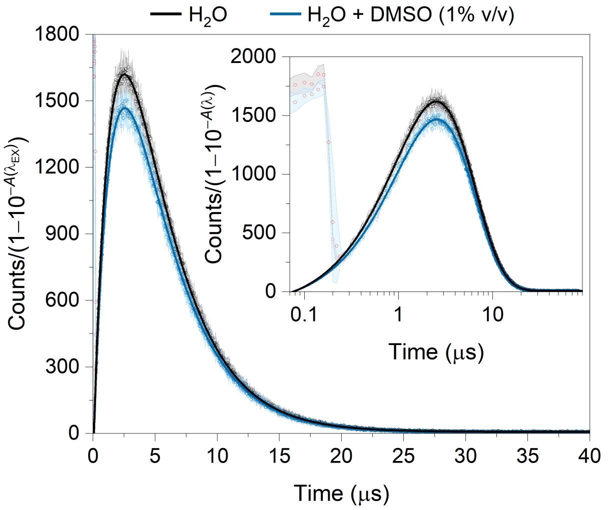

Figure S10. Effect of adding 1% v/v DMSO to an aqueous solution of phenalenone on the $^1O_2$ phosphorescence normalized by photon absorption. Fitting results: H_2O, SNR = 47 dB (222:1), *Abs*(400 nm) = 0.30, A = 1554 ± 11, τ_Δ = 3.68 ± 0.02 μs, τ_T = 1.67 ± 0.02 μs, t_0 = 0.08 ± 0.01 μs, y_0 = 3.2 ± 0.1, R^2 = 0.997, N = 7, Φ_Δ = 0.98 ± 0.08 (standard); H_2O + DMSO (1% v/v), SNR = 48

dB (244:1), Abs(400 nm) = 0.36, A = 1604 ± 15, τ_Δ = 3.72 ± 0.04 μs, τ_T = 1.70 ± 0.03 μs, t_0 = 0.08 ± 0.02 μs, y_0 = 2.9 ± 0.1, R^2 = 0.997, N = 7, Φ_Δ = 0.90 ± 0.07 (sample).

Note S6 – Rigorous Absorbance/Optical Density Measurement

Recording UV-Vis absorption spectra is considered a trivial experimental procedure, but small errors in the value of $A(\lambda)$ can have a major impact in the value of Φ_Δ, as this parameter influences the terms $1 - 10^{-A(\lambda)}$ (see Eq. (20)).[86]

Baseline and Offset. Correct instrumental artifacts (lamp-change breaks, noise $>10^{-4}$ from misalignment). Record spectra over a wide enough wavelength interval to reach a non-absorbing region (often at λ > 700 nm). Correct baselines by subtracting the average absorbance from ≥ 50 points in such regions. After correction, check for negative absorbance values caused by bubbles or mismatched blanks.

Linear Range. Above absorbance ~1.0–1.5, Beer–Lambert deviations occur due to stray light and detector saturation, distorting ε and Φ_Δ values. Below ~0.01–0.02, signals can be overwhelmed by the noise (especially in the near-IR/far-UV). Reliable quantitative data are therefore restricted to the 0.05–1.0 absorbance range, and analyte concentration or path length must be adjusted accordingly.[113] If excitation is carried out at a wavelength far from the absorption maximum, adjusting the sample to reach an absorbance of 0.05 at λ_{EX} may correspond to a much higher absorbance at λ_{max}. This indicates that the solution is in fact highly concentrated, increasing the risk of molecular aggregation and associated artifacts.

Cuvettes and Blanks. Quartz or fused silica cuvettes are required for wavelength <300 nm; plastic cuvettes often absorb UV and add background. Even with the right material, cuvettes must be clean and scratch-free, since contamination can mimic absorbance. Reference blanks must exactly match the sample matrix—solvent, salts, detergents, or co-solvents.

Temperature and Artifacts. Equilibrate solutions to room temperature before measurement to avoid bubble formation, which causes spectral noise. Gentle agitation can help reduce this effect. Particulates and aggregates scatter light, artificially inflating absorbance. For nanoparticle or dye aggregate systems, scattering corrections may be introduced with integrating spheres, dual-beam instruments, or modeled with λ^{-n} baselines.

Literature Extinction Coefficients. Reference $\varepsilon(\lambda)$ values (compiled in Table S1) exist for sensitizers like Rose Bengal, Methylene Blue, and phenalenone, and these should be used to cross-check both sample quality and instrument calibration. However, fresh spectra are still essential, since impurities, aggregation, or degradation alter absorption. This is especially critical for non-recrystallized ketones or photolabile dyes.

Reproducibility. Replicate spectra (≥ 3) should always be acquired and averaged, particularly in low-absorbance tail regions where small contributions matter. Good practice is <1% variance across replicates. Published work should also explicitly report the number of replicates and the associated variance.

Note S7 – Surplus Analysis

In cases where fitting convergence using Eq. (1) is poor due to mismatch in the early stages of the kinetic trace due to PS phosphorescence, complex STAs, or other sources of signal, surplus analysis is recommended. Fit Eq. (1) to the data in the time range from 7.0 μs to the end of the dataset and extrapolate the fitted curve back to t_0. Record the resulting α and τ_Δ. Subtract this fit from the raw data to obtain the residual (surplus) signal. Fit Eq. (1) to this surplus signal over the interval from t_0 to the end of the dataset and record the corresponding parameters α and τ_Δ. Use both the parameter sets as initial guesses for α, β, and τ_Δ, together with the value of τ_T determined,

when possible, by transient absorption or data fitting, to fit Eq. (S34) to the raw data across the entire time range. Ensure that all fits are performed using the same value of t_0.

$$
\begin{aligned}
S(t) = \alpha \, \frac{\tau_{\Delta,1}}{\tau_{\Delta,1} - \tau_{\mathrm{T}}} \left(e^{-(t-t_0)/\tau_{\Delta,1}} - e^{-(t-t_0)/\tau_{\mathrm{T}}} \right) + \\
\beta \frac{\tau_{\Delta,2}}{\tau_{\Delta,2} - \tau_{\mathrm{T}}} \left(e^{-(t-t_0)/\tau_{\Delta,2}} - e^{-(t-t_0)/\tau_{\mathrm{T}}} \right) + y_0, \quad t_0 \geq 0
\end{aligned}
\tag{S34}
$$

Note S8 – Aggregation of Photosensitizers

Aggregation is a critical factor often affecting the photophysical behavior of PSs. Many PSs, including Rose Bengal,[114] Methylene Blue,[115, 116] porphyrins, phthalocyanines, and BODIPYs, readily self-associate in aqueous media.[117] This typically results in shifts in absorption spectra, fluorescence quenching, and reduced Φ_Δ. Rose Bengal illustrates these problems clearly: above ~2 µmol L^{-1} it aggregates, leading to blue-shifted absorption and a non-linear dependence of $^1O_2$ generation on concentration; 10% and 23% reductions in τ_Δ have been measured at 1.7 and 3.4 µmol L^{-1}, respectively. Factors such as ionic strength (e.g., buffer concentration), pH, and lipid environments further modulate this effect, making Rose Bengal reliable as a reference standard only below 1–2 µmol L^{-1}.[114] Methylene Blue faces similar challenges in aqueous solution.[116, 118] In dilute solutions, Methylene Blue is mostly monomeric, but as its concentration increases (~1 µmol L^{-1} or above), the dimer form becomes significant. A new absorption band near 605–610 nm is observed when dimers form, alongside the monomer band at around 660 nm. Higher-order aggregates (trimers, tetramers, etc.) have been detected at still higher Methylene Blue concentrations. The threshold for trimer formation is reported to be in the range of 10 – 50 µmol L^{-1} at room temperature. The singlet excited-state lifetime of the Methylene Blue monomer is considerably longer (hundreds of picoseconds) compared with the dimer, whose singlet excited state decays in tens of picoseconds.[119] The shorter lifetime in the aggregate, due to faster

nonradiative decay and quenching, reduces the efficiency of intersystem crossing and, hence, the triplet-photosensitized formation of singlet oxygen.[116, 119] Consequently, as the Methylene Blue concentration increases, more dimer/aggregate forms are present and Φ_{Δ} decreases.

TROUBLESHOOTING

Problem	Description	Possible Solutions
Low Signal-to-Noise Ratio	The inherent weak phosphorescence emission of $^1O_2(^1\Delta_g)$ in H_2O can lead to low (< 5:1) signal-to-noise ratios.	• Modern detectors like thermoelectrically cooled NIR photomultiplier tubes offer improved sensitivity and temporal resolution, allowing reliable measurements in H_2O. Use high étendue detection to observe a bigger fraction of all emitted photons.<br>• Accumulate multiple signals until the SNR is > 5:1 to preserve the meaning of $\chi^2_{red.}$
PS Photobleaching	Absorption spectra before and after irradiation are different.	• If degradation is significant, consider using a quartz flow cuvette or reducing the excitation power.<br>• Split the measurement into consecutive measurements. Even at low SNR this allows one to identify most of the changes.
Artifacts and Contamination	Large spikes in the early stages of the signal can occur, often saturating detector electronics and obscuring the initial signal. This can be caused by scattered laser light or sensitizer fluorescence/phosphorescence.	• Spectral discrimination and a good housing.<br>Reduce scattering – most lasers contain a minute amount of NIR emission<br>Don't do analog measurements – counting electronics with sufficiently short dead time can reliably detect signals even after high count rates at the beginning

		• Employ a silicon cutoff filter (blocks light below 1050 nm) in combination with a 1270 nm interference filter. Make sure the filter is anti-reflection coated. The high refraction index of Si at 1270 nm (3.465) results in high reflection at the surfaces, thus losing about half the signal intensity.<br>• Be careful with long-pass filters because they may show dim emission that may be relevant in $^1O_2$ phosphorescence measurements.<br>• NIR background radiation (common with Nd:YAG lasers) can be problematic; the use of NIR blocking at each light source right from the start (e.g., Schott KG5) at every laser port is advisable.
Detector Limitations	The size of the germanium photodiode impacts both sensitivity and response time, representing a trade-off.	• Newer InGaAs diodes and NIR photomultiplier tubes offer higher gain and faster response.
Sample Impurities and Degradation	The τ_Δ is highly sensitive to impurities, especially those containing OH groups. Solutions stored for long periods can degrade.	• Ensure ultrapure water is used.<br>• Prepare solutions immediately before use and handle them under dim light.

Data Acquisition and Analysis Issues	Signal Authentication	• To rule out artifacts, the signal should disappear when oxygen is excluded (e.g., upon inert gas saturation) and its lifetime should decrease upon adding known $^1O_2$ quenchers, such as BSA, azide, 1,4-diazabicyclo[2.2.2]octane (DABCO), or β-carotene.<br>• Maintain constant $[O_2]$ (e.g. air-saturated buffer stirred or flowing) so that the pseudo-first-order assumption holds.
	Incorrect Kinetic Function Fitting	• If the sensitizer triplet lifetime and the $^1O_2$ lifetime are comparable ($\tau_T \approx \tau_\Delta$), using a simplified exponential decay function (tail fit) can lead to serious errors in estimating the initial amplitude parameter.<br>• It is recommendable to change pO_2 to achieve unequivocal results.
	Non-Linearity in α vs. excitation energy plots	• Measure with lower laser fluences if this is observed.
	Non-Linearity in α vs. fractional absorption plots	• Nonzero intercepts can indicate an absorbing impurity in the solvent or a mismatch between the laser and spectrophotometer wavelengths. Always run a "blank" of the solvent and calibrate the spectrophotometer.

	Concentration Effects	• In such cases, take the slope extrapolated to zero concentration as the true measure of Φ_Δ.<br>• Work at low PS concentrations (< μmol L^{-1} range) to avoid self-absorption/self-quenching.
Reproducibility problems		• Control temperature carefully.
Reference Photosensitizers	When a solvent lacks a well-established reference, determining Φ_Δ can be challenging.	• It is possible to use a reference in a different solvent, but solvent differences in the refractive index and radiative rate constant must be accounted for.<br>• Finding new single-molecule reference sensitizers with Φ_Δ values more comparable to PS (0.5–10%).
		Because absorption spectrometers have a certain wavelength accuracy, it is highly recommendable to choose a reference with a very similar slope of the absorption spectrum in the region of excitation. Otherwise, mispositioning of the absorption by only 0.5 nm may cause a big error in the absorption ratio between the standard and the sample.<br>Also, some sources are not spectrally narrow (like LEDs); here the slope must be considered as well, since the

		absorbed intensity is a convolution over the spectrum of the source.
Beam profile non-uniformity	Beam is too wide	• Use a homogenized beam or a small excitation volume.
Detector spectral roll-off	Amplitude distortions: not directly affecting rate constants, but scales I(t) incorrectly	• Calibrate the detector response.
Scattering and turbidity	Adds prompt spike; can mask early rise.	• Time-gated detection or polarization filtering.

REFERENCES

(1) Schlothauer, J.; Hackbarth, S.; Röder, B. A new benchmark for time-resolved detection of singlet oxygen luminescence - revealing the evolution of lifetime in living cells with low dose illumination. *Laser Physics Letters* **2009**, *6* (3), 216–221. DOI: 10.1002/lapl.200810116.

(2) Hackbarth, S.; Schlothauer, J.; Preuss, A.; Roder, B. New insights to primary photodynamic effects--Singlet oxygen kinetics in living cells. *J Photochem Photobiol B* **2010**, *98* (3), 173–179. DOI: 10.1016/j.jphotobiol.2009.11.013.

(3) Ghanadzadeh, A.; Zeini, A.; Kashef, A.; Moghadam, M. Concentration effect on the absorption spectra of oxazine1 and methylene blue in aqueous and alcoholic solutions. *Journal of Molecular Liquids* **2008**, *138* (1-3), 100–106. DOI: 10.1016/j.molliq.2007.09.005.

(4) Kikuchi, K.; Kokubun, H.; Kikuchi, M. Electron Transfer Reaction in the Triplet State. Role of Ferrocene as an Electron Donor. *Bulletin of the Chemical Society of Japan* **1975**, *48* (5), 1378–1381. DOI: 10.1246/bcsj.48.1378.

(5) Usui, Y. Determination of Quantum Yield of Singlet Oxygen Formation by Photosensitization. *Chemistry Letters* **1973**, *2* (7), 743–744. DOI: 10.1246/cl.1973.743.

(6) Merkel, P. B.; Kearns, D. R. Remarkable solvent effects on the lifetime of 1Δg oxygen. *Journal of the American Chemical Society* **1972**, *94* (3), 1029–1030. DOI: 10.1021/ja00758a071.

(7) Alarcon, E. I.; Gonzalez-Bejar, M.; Montes-Navajas, P.; Garcia, H.; Lissi, E. A.; Scaiano, J. C. Unexpected solvent isotope effect on the triplet lifetime of methylene blue associated to cucurbit[7]uril. *Photochem Photobiol Sci* **2012**, *11* (2), 269–273. DOI: 10.1039/c1pp05227f.

(8) Epstein, L. F.; Karush, F.; Rabinowitch, E. A Spectrophotometric Study of Thionine*. *Journal of the Optical Society of America* **1941**, *31* (1). DOI: 10.1364/josa.31.000077.

(9) Ferreira, M. I. C.; Harriman, A. Photoredox reactions of thionine. *Journal of the Chemical Society, Faraday Transactions 1: Physical Chemistry in Condensed Phases* **1977**, *73* (0). DOI: 10.1039/f19777301085.

(10) Ben Fradj, A.; lafi, R.; Gzara, L.; Hamzaoui, A. H.; Hafiane, A. Spectrophotometric study of the interaction of toluidine blue with poly (ammonium acrylate). *Journal of Molecular Liquids* **2014**, *194*, 110–114. DOI: 10.1016/j.molliq.2014.01.008.

(11) Mariño-Ocampo, N.; Reyes, J. S.; Günther, G.; Heyne, B.; Fuentealba, D. Thiol-reacting toluidine blue derivatives: Synthesis, photophysical properties and covalent conjugation with human serum albumin. *Dyes and Pigments* **2022**, *201*. DOI: 10.1016/j.dyepig.2022.110225.

(12) Querini-Sanguillen, W.; Otero-Gonzalez, J.; Garcia-Sanchez, M.; Zuniga-Nunez, D.; Gunther, G.; Miranda, M. L.; Castro-Perez, E.; Ramos, C.; Fuentealba, D.; Robinson-Duggon, J. Toluidine blue O demethylated photoproducts as type II photosensitizers. *Photochem Photobiol* **2025**, *101* (4), 1061–1071. DOI: 10.1111/php.14066.

(13) Ludvikova, L.; Fris, P.; Heger, D.; Sebej, P.; Wirz, J.; Klan, P. Photochemistry of rose bengal in water and acetonitrile: a comprehensive kinetic analysis. *Phys Chem Chem Phys* **2016**, *18* (24), 16266–16273. DOI: 10.1039/c6cp01710j.

(14) Redmond, R. W. ENHANCEMENT OF THE SENSITIVITY OF RADIATIVE and NON-RADIATIVE DETECTION TECHNIQUES IN THE STUDY OF PHOTOSENSITIZATION BY WATER SOLUBLE SENSITIZERS USING A REVERSE MICELLE SYSTEMS. *Photochemistry photobiology*

1991, *54* (4), 547–556.

(15) Scholz, M.; Dedic, R.; Breitenbach, T.; Hala, J. Singlet oxygen-sensitized delayed fluorescence of common water-soluble photosensitizers. *Photochem Photobiol Sci* **2013**, *12* (10), 1873–1884. DOI: 10.1039/c3pp50170a.

(16) Penzkofer, A.; Simmel, M.; Riedl, D. Room temperature phosphorescence lifetime and quantum yield of erythrosine B and rose bengal in aerobic alkaline aqueous solution. *Journal of Luminescence* **2012**, *132* (4), 1055–1062. DOI: 10.1016/j.jlumin.2011.12.030.

(17) Gemmell, N. R.; McCarthy, A.; Kim, M. M.; Veilleux, I.; Zhu, T. C.; Buller, G. S.; Wilson, B. C.; Hadfield, R. H. A compact fiber-optic probe-based singlet oxygen luminescence detection system. *J Biophotonics* **2017**, *10* (2), 320–326. DOI: 10.1002/jbio.201600078.

(18) Kearns, D. R.; Hollins, R. A.; Khan, A. U.; Radlick, P. Evidence for the participation of 1G + and 1.DELTA.g oxygen in dye-sensitized photooxygenation reactions. II. *Journal of the American Chemical Society* **1967**, *89* (21), 5456–5457. DOI: 10.1021/ja00997a030.

(19) Penzkofer, A.; Beidoun, A.; Daiber, M. Intersystem-crossing and excited-state absorption in eosin Y solutions determined by picosecond double pulse transient absorption measurements. *Journal of Luminescence* **1992**, *51* (6), 297–314. DOI: 10.1016/0022-2313(92)90059-i.

(20) Zakharova, G. V.; Korobov, V. E.; Shabalov, V. V.; Chibisov, A. K. Quenching of rhodamine-6G triplet state by inorganic ions in aqueous solutions. *Journal of Applied Spectroscopy* **1983**, *39* (1), 765–768. DOI: 10.1007/bf00662817.

(21) Korobov, V. E.; Shubin, V. V.; Chibisov, A. K. Triplet state of rhodamine dyes and its role in production of intermediates. *Chemical Physics Letters* **1977**, *45* (3), 498–501. DOI: 10.1016/0009-2614(77)80074-2.

(22) Haimerl, J. M.; Ghosh, I.; Konig, B.; Lupton, J. M.; Vogelsang, J. Chemical Photocatalysis with Rhodamine 6G: Investigation of Photoreduction by Simultaneous Fluorescence Correlation

Spectroscopy and Fluorescence Lifetime Measurements. *J Phys Chem B* **2018**, *122* (47), 10728–10735. DOI: 10.1021/acs.jpcb.8b08615.

(23) Zhao, H.; Takano, Y.; Sasikumar, D.; Miyatake, Y.; Biju, V. Excitation-Wavelength-Dependent Functionalities of Temporally Controlled Sensing and Generation of Singlet Oxygen by a Photoexcited State Engineered Rhodamine 6G-Anthracene Conjugate. *Chemistry* **2022**, *28* (71), e202202014. DOI: 10.1002/chem.202202014.

(24) Soep, B.; Kellmann, A.; Martin, M.; Lindqvist, L. Study of triplet quantum yields using a tunable dye laser. *Chemical Physics Letters* **1972**, *13* (3), 241–244. DOI: 10.1016/0009-2614(72)85051-6.

(25) Taniguchi, M.; Lindsey, J. S. Database of Absorption and Fluorescence Spectra of >300 Common Compounds for use in PhotochemCAD. *Photochem Photobiol* **2018**, *94* (2), 290–327. DOI: 10.1111/php.12860.

(26) Redmond, R. W.; Gamlin, J. N. A Compilation of Singlet Oxygen Yields from Biologically Relevant Molecules. *Photochemistry and Photobiology* **1999**, *70* (4), 391–475. DOI: 10.1111/j.1751-1097.1999.tb08240.x.

(27) Gollnick, K.; Franken, T.; Fouda, M. F. R.; Paur, H. R.; Held, S. Merbromin (mercurochrome) and other xanthene dyes: Quantum yields of triplet sensitizer generation and singlet oxygen formation in alcoholic solutions. *Journal of Photochemistry and Photobiology B: Biology* **1992**, *12* (1), 57–81. DOI: 10.1016/1011-1344(92)85018-p.

(28) Karlsson, J. K. G.; Woodford, O. J.; Al-Aqar, R.; Harriman, A. Effects of Temperature and Concentration on the Rate of Photobleaching of Erythrosine in Water. *J Phys Chem A* **2017**, *121* (45), 8569–8576. DOI: 10.1021/acs.jpca.7b06440.

(29) Bowers, P. G.; Porter, G. Triplet state quantum yields for some arom atic hydrocarbons and xanthene dyes in dilute solution. *Proceedings of the Royal Society of London. Series A. Mathematical and Physical Sciences* **1967**, *299* (1458), 348–353. DOI: 10.1098/rspa.1967.0141.

(30) Chartier, A.; Georges, J.; Mermet, J. M. Abnormal signals in thermal lens spectrophotometry: Determination of the triplet lifetime of erythrosine. *Spectrochimica Acta Part A: Molecular Spectroscopy* **1990**, *46* (12), 1737–1742. DOI: 10.1016/0584-8539(90)80245-t.

(31) Batistela, V. R.; Pellosi, D. S.; de Souza, F. D.; da Costa, W. F.; de Oliveira Santin, S. M.; de Souza, V. R.; Caetano, W.; de Oliveira, H. P.; Scarminio, I. S.; Hioka, N. pKa determinations of xanthene derivates in aqueous solutions by multivariate analysis applied to UV-Vis spectrophotometric data. *Spectrochim Acta A Mol Biomol Spectrosc* **2011**, *79* (5), 889–897. DOI: 10.1016/j.saa.2011.03.027.

(32) Mansha, A.; Grampp, G.; Landgraf, S. Photoinduced electron transfer between triplet erythrosin dianion and highly charged ionic quenchers. *Monatshefte für Chemie - Chemical Monthly* **2010**, *142* (1), 11–17. DOI: 10.1007/s00706-010-0414-1.

(33) Gandin, E.; Lion, Y.; Vandevorst, A. Quantum Yield of Singlet Oxygen Production by Xanthene Derivatives. *Photochemistry and Photobiology* **1983**, *37* (3), 271–278. DOI: 10.1111/j.1751-1097.1983.tb04472.x.

(34) Montalti, M.; Credi, A.; Prodi, L.; Gandolfi, M. T. *Handbook of Photochemistry*; CRC press, 2006. DOI: 10.1201/9781420015195.

(35) Shimizu, R.; Yagi, M.; Kikuchi, A. Suppression of riboflavin-sensitized singlet oxygen generation by l-ascorbic acid, 3-O-ethyl-l-ascorbic acid and Trolox. *J Photochem Photobiol B* **2019**, *191*, 116–122. DOI: 10.1016/j.jphotobiol.2018.12.012.

(36) Chambers, R. W.; Kearns, D. R. Triplet states of some common photosensitizing dyes. *Photochem Photobiol* **1969**, *10* (3), 215–219. DOI: 10.1111/j.1751-1097.1969.tb05681.x.

(37) Knak, A.; Regensburger, J.; Maisch, T.; Baumler, W. Exposure of vitamins to UVB and UVA radiation generates singlet oxygen. *Photochem Photobiol Sci* **2014**, *13* (5), 820–829. DOI: 10.1039/c3pp50413a.

(38) Oliveros, E.; Suardi-Murasecco, P.; Aminian-Saghafi, T.; Braun, A. M.; Hansen, H. J. 1H-Phenalen-1-one: Photophysical Properties and Singlet-Oxygen Production. *Helvetica Chimica Acta* **1991**, *74* (1), 79–90. DOI: 10.1002/hlca.19910740110.

(39) Schmidt, R.; Tanielian, C.; Dunsbach, R.; Wolff, C. Phenalenone, a universal reference compound for the determination of quantum yields of singlet oxygen O2(1Δg) sensitization. *Journal of Photochemistry and Photobiology A: Chemistry* **1994**, *79* (1-2), 11–17. DOI: 10.1016/1010-6030(93)03746-4.

(40) Flors, C.; Nonell, S. On the Phosphorescence of 1H-Phenalen-1-one. *Helvetica Chimica Acta* **2001**, *84* (9). DOI: 10.1002/1522-2675(20010919)84:9<2533::Aid-hlca2533>3.0.Co;2-l.

(41) Pasternack, R. F.; Gibbs, E. J.; Villafranca, J. J. Interactions of porphyrins with nucleic acids. *Biochemistry* **1983**, *22* (10), 2406–2414. DOI: 10.1021/bi00279a016.

(42) Borissevitch, I. E.; Ferreira, L. P.; Gonçalves, P. J.; Amado, A. M.; Schlothauer, J. C.; Baptista, M. S. Quenching of meso-tetramethylpyridyl porphyrin excited triplet state by inorganic salts: Exciplex formation. *Journal of Photochemistry and Photobiology A: Chemistry* **2018**, *367*, 156–161. DOI: 10.1016/j.jphotochem.2018.08.020.

(43) Kalyanasundaram, K.; Neumann-Spallart, M. Photophysical and redox properties of water-soluble porphyrins in aqueous media. *The Journal of Physical Chemistry* **2002**, *86* (26), 5163–5169. DOI: 10.1021/j100223a022.

(44) Angeli, N. G.; Lagorio, M. G.; Román, E. A. S.; Dicelio, L. E. Meso-Substituted Cationic Porphyrins of Biological Interest. Photophysical and Physicochemical Properties in Solution and Bound to Liposomes ¶. *Photochemistry and Photobiology* **2007**, *72* (1), 49–56. DOI: 10.1562/0031-8655(2000)0720049mscpob2.0.Co2.

(45) Eisenstein, K. K.; Wang, J. H. Conversion of Light to Chemical Free Energy. *Journal of Biological Chemistry* **1969**, *244* (7), 1720–1728. DOI: 10.1016/s0021-9258(18)91743-6.

(46) Lambert, C. R.; Reddi, E.; Spikes, J. D.; Rodgers, M. A.; Jori, G. The effects of porphyrin structure and aggregation state on photosensitized processes in aqueous and micellar media. *Photochem Photobiol* **1986**, *44* (5), 595–601. DOI: 10.1111/j.1751-1097.1986.tb04714.x.

(47) Murgia, S. M.; Pasqua, A.; Poletti, A. Laser photolysis study of the hematoporphyrin IX—ℓ-tryptophan system in solvent mixtures at different polarity. *Chemical Physics Letters* **1983**, *98* (2), 179–183. DOI: 10.1016/0009-2614(83)87124-3.

(48) Spikes, J. D.; Bommer, J. C. Photosensitizing properties of mono-L-aspartyl chlorin e6 (NPe6): a candidate sensitizer for the photodynamic therapy of tumors. *J Photochem Photobiol B* **1993**, *17* (2), 135–143. DOI: 10.1016/1011-1344(93)80006-u.

(49) Parra, G. G.; Correa, D. S.; Silveira-Alves, E., Jr.; Almeida, L. M.; Souza, M. A. R.; De Boni, L.; Misoguti, L.; Mendonca, C. R.; Zilio, S. C.; Barbosa Neto, N. M.; et al. Effects of meso-tetrakis (4-sulfonatophenyl) porphyrin (TPPS(4)) aggregation on its spectral and kinetic characteristics and singlet oxygen production. *Spectrochim Acta A Mol Biomol Spectrosc* **2021**, *261*, 120063. DOI: 10.1016/j.saa.2021.120063.

(50) Aggarwal, L. P.; Borissevitch, I. E. On the dynamics of the TPPS4 aggregation in aqueous solutions: successive formation of H and J aggregates. *Spectrochim Acta A Mol Biomol Spectrosc* **2006**, *63* (1), 227–233. DOI: 10.1016/j.saa.2005.05.009.

(51) Mostafa, G. A. E.; Mahajumi, A. S.; AlRabiah, H.; Kadi, A. A.; Lu, Y.; Rahman, A. Synthesis and Photophysical Properties of Fluorescein Esters as Potential Organic Semiconductor Materials. *J Fluoresc* **2021**, *31* (5), 1489–1502. DOI: 10.1007/s10895-021-02789-y.

(52) Lutkus, L. V.; Rickenbach, S. S.; McCormick, T. M. Singlet oxygen quantum yields determined by oxygen consumption. *Journal of Photochemistry and Photobiology A: Chemistry* **2019**, *378*, 131–135. DOI: 10.1016/j.jphotochem.2019.04.029.

(53) Song, L.; Varma, C. A.; Verhoeven, J. W.; Tanke, H. J. Influence of the triplet excited state on the photobleaching kinetics of fluorescein in microscopy. *Biophys J* **1996**, *70* (6), 2959–2968. DOI: 10.1016/S0006-3495(96)79866-1.

(54) Wolcan, E. On the origins of the absorption spectroscopy of pterin and Re(CO)3(pterin)(H2O) aqueous solutions. A combined theoretical and experimental study. *Spectrochim Acta A Mol Biomol Spectrosc* **2014**, *129*, 173–183. DOI: 10.1016/j.saa.2014.03.022.

(55) Chahidi, C.; Aubailly, M.; Momzikoff, A.; Bazin, M.; Santus, R. Photophysical and Photosensitizing Properties of 2-Amino-4 Pteridinone: A Natural Pigment. *Photochemistry and Photobiology* **2008**, *33* (5), 641–649. DOI: 10.1111/j.1751-1097.1981.tb05470.x.

(56) Denofrio, M. P.; Ogilby, P. R.; Thomas, A. H.; Lorente, C. Selective quenching of triplet excited states of pteridines. *Photochem Photobiol Sci* **2014**, *13* (7), 1058–1065. DOI: 10.1039/c4pp00079j.

(57) Thomas, A. H.; Lorente, C.; Capparelli, A. L.; Martinez, C. G.; Braun, A. M.; Oliveros, E. Singlet oxygen (1Δg) production by pterin derivatives in aqueous solutions. *Photochem. Photobiol. Sci.* **2003**, *2* (3), 245–250. DOI: 10.1039/B209993D.

(58) Lorente, C.; Thomas, A. H. Photophysics and photochemistry of pterins in aqueous solution. *Acc Chem Res* **2006**, *39* (6), 395–402. DOI: 10.1021/ar050151c.

(59) Cui, S.; Guo, X.; Wang, S.; Wei, Z.; Huang, D.; Zhang, X.; Zhu, T. C.; Huang, Z. Singlet Oxygen in Photodynamic Therapy. *Pharmaceuticals (Basel)* **2024**, *17* (10). DOI: 10.3390/ph17101274.

(60) Sekkat, N.; van den Bergh, H.; Nyokong, T.; Lange, N. Like a bolt from the blue: phthalocyanines in biomedical optics. *Molecules* **2011**, *17* (1), 98–144. DOI: 10.3390/molecules17010098.

(61) Horne, J. E.; Goldblatt, C.; Kump, L. An early origin of oxygenic photosynthesis delays the Great Oxidation. *Philosophical Transactions of the Royal Society B: Biological Sciences* **2025**, *380* (1931). DOI: 10.1098/rstb.2024.0094.

(62) Borden, W. T.; Hoffmann, R.; Stuyver, T.; Chen, B. Dioxygen: What Makes This Triplet Diradical Kinetically Persistent? *J Am Chem Soc* **2017**, *139* (26), 9010–9018. DOI: 10.1021/jacs.7b04232.

(63) The reaction between ground state molecules (closed-shell singlets) and molecular oxygen are forbidden by energy and spin conservation, though its reaction with carbon-centered radicals (open-shell, doublet multiplicity) are spin allowed. Consequently, combustion proceeds via thermally or photochemically initiated radical chain mechanisms.

(64) Bregnhoj, M.; Westberg, M.; Minaev, B. F.; Ogilby, P. R. Singlet Oxygen Photophysics in Liquid Solvents: Converging on a Unified Picture. *Acc Chem Res* **2017**, *50* (8), 1920–1927. DOI: 10.1021/acs.accounts.7b00169.

(65) Wessels, J. M.; Rodgers, M. A. J. Effect of Solvent Polarizability on the Forbidden 1.DELTA.g .fwdarw. 3.SIGMA.g- Transition in Molecular Oxygen: A Fourier Transform Near-Infrared Luminescence Study. *The Journal of Physical Chemistry* **1995**, *99* (49), 17586–17592. DOI: 10.1021/j100049a019.

(66) Adam, W.; Kazakov, D. V.; Kazakov, V. P. Singlet-oxygen chemiluminescence in peroxide reactions. *Chem Rev* **2005**, *105* (9), 3371–3387. DOI: 10.1021/cr0300035.

(67) Ghogare, A. A.; Greer, A. Using Singlet Oxygen to Synthesize Natural Products and Drugs. *Chem Rev* **2016**, *116* (17), 9994–10034. DOI: 10.1021/acs.chemrev.5b00726.

(68) Badger, R. M.; Wright, A. C.; Whitlock, R. F. Absolute Intensities of the Discrete and Continuous Absorption Bands of Oxygen Gas at 1.26 and 1.065 μ and the Radiative Lifetime of the 1Δg State of Oxygen. *The Journal of Chemical Physics* **1965**, *43* (12), 4345–4350. DOI: 10.1063/1.1696694.

(69) Minaev, B. F.; Ågren, H. Collision-induced b1Σg+–a1 Δg, b1Σg+–X3 Σg- and a1Δg–X3Σg - transition probabilities in molecular oxygen. *Journal of the Chemical Society, Faraday Transactions* **1997**, *93* (13), 2231–2239. DOI: 10.1039/a607263a.

(70) Schweitzer, C.; Schmidt, R. Physical mechanisms of generation and deactivation of singlet oxygen. *Chem Rev* **2003**, *103* (5), 1685–1757. DOI: 10.1021/cr010371d.

(71) Baier, J.; Fuss, T.; Pollmann, C.; Wiesmann, C.; Pindl, K.; Engl, R.; Baumer, D.; Maier, M.; Landthaler, M.; Baumler, W. Theoretical and experimental analysis of the luminescence signal of singlet oxygen for different photosensitizers. *J Photochem Photobiol B* **2007**, *87* (3), 163–173. DOI: 10.1016/j.jphotobiol.2007.02.006.

(72) Maisch, T.; Baier, J.; Franz, B.; Maier, M.; Landthaler, M.; Szeimies, R. M.; Baumler, W. The role of singlet oxygen and oxygen concentration in photodynamic inactivation of bacteria. *Proc Natl Acad Sci U S A* **2007**, *104* (17), 7223–7228. DOI: 10.1073/pnas.0611328104.

(73) Boix-Garriga, E.; Rodríguez-Amigo, B.; Planas, O.; Nonell, S. Properties of singlet oxygen. *Singlet Oxygen: Applications in Biosciences and Nanosciences* **2016**, *1*, 23–46.

(74) Minaev, B. F. Spin-orbit coupling mechanism of singlet oxygen a1Δg quenching by solvent vibrations. *Chemical Physics* **2017**, *483-484*, 84–95. DOI: 10.1016/j.chemphys.2016.11.012.

(75) Ansari, I. M.; Heller, E. R.; Trenins, G.; Richardson, J. O. Heavy-atom tunnelling in singlet oxygen deactivation predicted by instanton theory with branch-point singularities. *Nat Commun* **2024**, *15* (1), 4335. DOI: 10.1038/s41467-024-48463-2.

(76) Ogilby, P. R.; Foote, C. S. Chemistry of singlet oxygen. 42. Effect of solvent, solvent isotopic substitution, and temperature on the lifetime of singlet molecular oxygen (1.DELTA.g). *Journal of the American Chemical Society* **1983**, *105* (11), 3423–3430. DOI: 10.1021/ja00349a007.

(77) Wilkinson, F.; Helman, W. P.; Ross, A. B. Quantum Yields for the Photosensitized Formation of the Lowest Electronically Excited Singlet State of Molecular Oxygen in Solution. *Journal of Physical and Chemical Reference Data* **1993**, *22* (1), 113–262. DOI: 10.1063/1.555934.

(78) Wilkinson, F.; Helman, W. P.; Ross, A. B. Rate Constants for the Decay and Reactions of the Lowest Electronically Excited Singlet State of Molecular Oxygen in Solution. An Expanded and Revised Compilation. *Journal of Physical and Chemical Reference Data* **1995**, *24* (2), 663–677. DOI: 10.1063/1.555965.

(79) Mosinger, J.; Lang, K.; Hostomsky, J.; Franc, J.; Sykora, J.; Hof, M.; Kubat, P. Singlet oxygen imaging in polymeric nanofibers by delayed fluorescence. *J Phys Chem B* **2010**, *114* (48), 15773–15779. DOI: 10.1021/jp105789p.

(80) DeRosa, M.; Crutchley, R. J. Photosensitized singlet oxygen and its applications. *Coordination Chemistry Reviews* **2002**, *233-234*, 351–371. DOI: 10.1016/s0010-8545(02)00034-6.

(81) Braslavsky, S. E. Glossary of terms used in photochemistry, 3rd edition (IUPAC Recommendations 2006). *Pure and Applied Chemistry* **2007**, *79* (3), 293–465. DOI: 10.1351/pac200779030293.

(82) Here, we use the premise of Klán and Wirz that the quantum yield of a single-step reaction is equal to its efficiency (Φi = ηi) and the quantum yields of a multi-step process is equal to the product of the efficiencies of all steps involved in the process, connecting reaction rate constants (and corresponding lifetimes) with quantum yields.

(83) Klán, P.; Wirz, J. *Photochemistry of Organic Compounds: From Concepts to Practice*; John Wiley & Sons, Ltd, 2009.

(84) Nonell, S.; Braslavsky, S. E. Time-resolved singlet oxygen detection. *Methods Enzymol* **2000**, *319*, 37–49. DOI: 10.1016/s0076-6879(00)19006-8.

(85) Erickson, P. R.; Moor, K. J.; Werner, J. J.; Latch, D. E.; Arnold, W. A.; McNeill, K. Singlet Oxygen Phosphorescence as a Probe for Triplet-State Dissolved Organic Matter Reactivity. *Environ Sci Technol* **2018**, *52* (16), 9170–9178. DOI: 10.1021/acs.est.8b02379.

(86) Ossola, R.; Jonsson, O. M.; Moor, K.; McNeill, K. Singlet Oxygen Quantum Yields in Environmental Waters. *Chem Rev* **2021**, *121* (7), 4100–4146. DOI: 10.1021/acs.chemrev.0c00781.

(87) Bregnhoj, M.; Thorning, F.; Ogilby, P. R. Singlet Oxygen Photophysics: From Liquid Solvents to Mammalian Cells. *Chem Rev* **2024**, *124* (17), 9949–10051. DOI: 10.1021/acs.chemrev.4c00105.

(88) Stracke, F.; Heupel, M.; Thiel, E. Singlet molecular oxygen photosensitized by Rhodamine dyes: correlation with photophysical properties of the sensitizers. *Journal of Photochemistry and Photobiology A: Chemistry* **1999**, *126* (1-3), 51–58. DOI: 10.1016/s1010-6030(99)00123-9.

(89) Baumer, D.; Maier, M.; Engl, R.; Markus Szeimies, R.; Bäumler, W. Singlet oxygen generation by 9-acetoxy-2,7,12,17-tetrakis-(β-methoxyethyl)-porphycene (ATMPn) in solution. *Chemical Physics* **2002**, *285* (2-3), 309–318. DOI: 10.1016/s0301-0104(02)00806-6.

(90) Scurlock, R. D.; Ogilby, P. R. Effect of solvent on the rate constant for the radiative deactivation of singlet molecular oxygen (1ΔgO2). *The Journal of Physical Chemistry* **1987**, *91* (17), 4599–4602. DOI: 10.1021/j100301a034.

(91) Bacellar, I. O. L.; Cordeiro, R. M.; Mahling, P.; Baptista, M. S.; Roder, B.; Hackbarth, S. Oxygen distribution in the fluid/gel phases of lipid membranes. *Biochim Biophys Acta Biomembr* **2019**, *1861* (4), 879–886. DOI: 10.1016/j.bbamem.2019.01.019.

(92) Hackbarth, S.; Roder, B. Singlet oxygen luminescence kinetics in a heterogeneous environment - identification of the photosensitizer localization in small unilamellar vesicles. *Photochem Photobiol Sci* **2015**, *14* (2), 329–334. DOI: 10.1039/c4pp00229f.

(93) El Seoud, O. A.; Baader, W. J.; Bastos, E. L. Practical Chemical Kinetics in Solution. In *Encyclopedia of Physical Organic Chemistry, 5 Volume Set*, 2016; pp 1–68.

(94) Bravo-Diaz, C.; Losada-Barreiro, S.; Santos, J. G.; Aliaga, M. E. Nonlinear Regression Analyses: Principles and Comparisons Between Experimental and Fitting Data. In *From Experimental Kinetic Data to Reaction Mechanisms*, Physical Chemistry in Action, 2025; pp 227–238.

(95) Hackbarth, S.; Pfitzner, M.; Pohl, J.; Röder, B. *Singlet Oxygen Detection and Imaging*; Morgan & Claypool, 2021. DOI: 10.1007/978-3-031-02391-0.

(96) Wilson, S. T.; Fargher, S.; Foster, R.; Malek, M.; Needham, M.; Scarff, A.; Smith, G. D. Characterisation of the temperature-dependent dark rate of Hamamatsu R7081-100 10”

photomultiplier tubes. *Journal of Instrumentation* **2023**, *18* (08). DOI: 10.1088/1748-0221/18/08/p08017.

(97) Qian, X.; Jiang, W.; Elsharabasy, A.; Deen, M. J. Modeling for Single-Photon Avalanche Diodes: State-of-the-Art and Research Challenges. *Sensors (Basel)* **2023**, *23* (7). DOI: 10.3390/s23073412.

(98) Benavides, J. M.; Webb, R. H. Optical characterization of ultrabright LEDs. *Appl Opt* **2005**, *44* (19), 4000–4003. DOI: 10.1364/ao.44.004000.

(99) Niedre, M.; Patterson, M. S.; Wilson, B. C. Direct Near-infrared Luminescence Detection of Singlet Oxygen Generated by Photodynamic Therapy in Cells In Vitro and Tissues In Vivo¶. *Photochemistry and Photobiology* **2002**, *75* (4), 382–391. DOI: 10.1562/0031-8655(2002)0750382dnildo2.0.Co2.

(100) Pfitzner, M.; Schlothauer, J. C.; Bastien, E.; Hackbarth, S.; Bezdetnaya, L.; Lassalle, H. P.; Roder, B. Prospects of in vivo singlet oxygen luminescence monitoring: Kinetics at different locations on living mice. *Photodiagnosis Photodyn Ther* **2016**, *14*, 204–210. DOI: 10.1016/j.pdpdt.2016.03.002.

(101) Looft, A.; Pfitzner, M.; Preuss, A.; Roder, B. In vivo singlet molecular oxygen measurements: Sensitive to changes in oxygen saturation during PDT. *Photodiagnosis Photodyn Ther* **2018**, *23*, 325–330. DOI: 10.1016/j.pdpdt.2018.07.006.

(102) Firey, P. A.; Ford, W. E.; Sounik, J. R.; Kenney, M. E.; Rodgers, M. A. J. Silicon naphthalocyanine triplet state and oxygen. A reversible energy-transfer reaction. *Journal of the American Chemical Society* **1988**, *110* (23), 7626–7630. DOI: 10.1021/ja00231a007.

(103) Scholz, M.; Dedic, R.; Valenta, J.; Breitenbach, T.; Hala, J. Real-time luminescence microspectroscopy monitoring of singlet oxygen in individual cells. *Photochem Photobiol Sci* **2014**, *13* (8), 1203–1212. DOI: 10.1039/c4pp00121d.

(104) Stroop, S. D.; Conca, D. M.; Lundgard, R. P.; Renz, M. E.; Peabody, L. M.; Leigh, S. D. Photosensitizers form in histidine buffer and mediate the photodegradation of a monoclonal antibody. *J Pharm Sci* **2011**, *100* (12), 5142–5155. DOI: 10.1002/jps.22714.

(105) Gerlach, T.; Nugroho, D. L.; Rother, D. The Effect of Visible Light on the Catalytic Activity of PLP-Dependent Enzymes. *ChemCatChem* **2021**, *13* (10), 2398–2406. DOI: 10.1002/cctc.202100163.

(106) Ahmad, I.; Anwar, Z.; Iqbal, K.; Ali, S. A.; Mirza, T.; Khurshid, A.; Khurshid, A.; Arsalan, A. Effect of acetate and carbonate buffers on the photolysis of riboflavin in aqueous solution: a kinetic study. *AAPS PharmSciTech* **2014**, *15* (3), 550–559. DOI: 10.1208/s12249-013-0067-6.

(107) Goncalves, L. C. P.; Mansouri, H. R.; Bastos, E. L.; Abdellah, M.; Fadiga, B. S.; Sa, J.; Rudroff, F.; Mihovilovic, M. D. Morpholine-based buffers activate aerobic photobiocatalysis via spin correlated ion pair formation. *Catal Sci Technol* **2019**, *9* (6), 1365–1371. DOI: 10.1039/c8cy02524j.

(108) Gonçalves, L. C. P.; Mansouri, H. R.; PourMehdi, S.; Abdellah, M.; Fadiga, B. S.; Bastos, E. L.; Sá, J.; Mihovilovic, M. D.; Rudroff, F. Boosting photobioredox catalysis by morpholine electron donors under aerobic conditions. *Catalysis Science & Technology* **2019**, *9* (10), 2682–2688. DOI: 10.1039/c9cy00496c.

(109) Lee, H.; Hong, J. Modulation of Photosensitizing Responses in Cell Culture Environments by Different Medium Components. *Int J Mol Sci* **2024**, *25* (18). DOI: 10.3390/ijms251810016.

(110) Muller, A.; Preuss, A.; Roder, B. Photodynamic inactivation of Escherichia coli - Correlation of singlet oxygen kinetics and phototoxicity. *J Photochem Photobiol B* **2018**, *178*, 219–227. DOI: 10.1016/j.jphotobiol.2017.11.017.

(111) Silva, P. L.; Bastos, E. L.; El Seoud, O. A. Solvation in binary mixtures of water and polar aprotic solvents: theoretical calculations of the concentrations of solvent-water hydrogen-bonded species and application to thermosolvatochromism of polarity probes. *J Phys Chem B* **2007**, *111* (22), 6173–6180. DOI: 10.1021/jp068596l.

(112) LeBel, R. G.; Goring, D. A. I. Density, Viscosity, Refractive Index, and Hygroscopicity of Mixtures of Water and Dimethyl Sulfoxide. *Journal of Chemical & Engineering Data* **1962**, *7* (1), 100–101. DOI: 10.1021/je60012a032.

(113) Fery-Forgues, S.; Lavabre, D. Are Fluorescence Quantum Yields So Tricky to Measure? A Demonstration Using Familiar Stationery Products. *Journal of Chemical Education* **1999**, *76* (9). DOI: 10.1021/ed076p1260.

(114) Mokrzynski, K.; Szewczyk, G. The (un)known issue with using rose bengal as a standard of singlet oxygen photoproduction. *Photochem Photobiol* **2025**, *101* (3), 546–549. DOI: 10.1111/php.14030.

(115) Santin, L. R. R.; dos Santos, S. C.; Novo, D. L. R.; Bianchini, D.; Gerola, A. P.; Braga, G.; Caetano, W.; Moreira, L. M.; Bastos, E. L.; Romani, A. P.; et al. Study between solvatochromism and steady-state and time-resolved fluorescence measurements of the Methylene blue in binary mixtures. *Dyes and Pigments* **2015**, *119*, 12–21. DOI: 10.1016/j.dyepig.2015.03.004.

(116) Thompson, B. J.; Kumar, A.; Huxter, V. M. Concentration-dependent aggregation of methylene blue acting as a photoredox catalyst. *Phys Chem Chem Phys* **2024**, *26* (29), 19900–19907. DOI: 10.1039/d4cp02026j.

(117) Lan, M.; Zhao, S.; Liu, W.; Lee, C. S.; Zhang, W.; Wang, P. Photosensitizers for Photodynamic Therapy. *Adv Healthc Mater* **2019**, *8* (13), e1900132. DOI: 10.1002/adhm.201900132.

(118) Medhi, D.; Moral, R.; Paul, S.; Hazarika, S. From monomer to pentamer: solvent-driven aggregation pathways of methylene blue investigated via spectroscopy and molecular dynamics simulations. *Journal of Photochemistry and Photobiology A: Chemistry* **2026**, *474*. DOI: 10.1016/j.jphotochem.2025.116929.

(119) Fernandez-Perez, A.; Marban, G. Visible Light Spectroscopic Analysis of Methylene Blue in Water; What Comes after Dimer? *ACS Omega* **2020**, *5* (46), 29801–29815. DOI: 10.1021/acsomega.0c03830.
